# Comparison of three scoring methods using the FDA-approved 22C3 immunohistochemistry assay to evaluate PD-L1 expression in breast cancer and their association with clinicopathologic factors

**DOI:** 10.1186/s13058-020-01303-9

**Published:** 2020-06-23

**Authors:** Hua Guo, Qingqing Ding, Yun Gong, Michael Z. Gilcrease, Min Zhao, Jun Zhao, Dawen Sui, Yun Wu, Hui Chen, Hui Liu, Jinxia Zhang, Erika Resetkova, Stacy L. Moulder, Wei-Lien Wang, Lei Huo

**Affiliations:** 1 Department of Pathology, The University of Texas MD Anderson Cancer Center, Unit 85, 1515 Holcombe Blvd, Houston, TX 77030 USA; 2 Department of Biostatistics, The University of Texas MD Anderson Cancer Center, Houston, TX USA; 3 Department of Breast Medical Oncology, The University of Texas MD Anderson Cancer Center, Houston, TX USA

**Keywords:** PD-L1, 22C3, Immunohistochemistry, Scoring methods, Breast cancer, Tumor-infiltrating lymphocytes, Race/ethnicity

## Abstract

**Background:**

In the evaluation of PD-L1 expression to select patients for anti-PD-1/PD-L1 treatment, uniform guidelines that account for different immunohistochemistry assays, different cell types and different cutoff values across tumor types are lacking. Data on how different scoring methods compare in breast cancer are scant.

**Methods:**

Using FDA-approved 22C3 diagnostic immunohistochemistry assay, we retrospectively evaluated PD-L1 expression in 496 primary invasive breast tumors that were not exposed to anti-PD-1/PD-L1 treatment and compared three scoring methods (TC: invasive tumor cells; IC: tumor-infiltrating immune cells; TCIC: a combination of tumor cells and immune cells) in expression frequency and association with clinicopathologic factors.

**Results:**

In the entire cohort, positive PD-L1 expression was observed in 20% of patients by TCIC, 16% by IC, and 10% by TC, with a concordance of 87% between the three methods. In the triple-negative breast cancer patients, positive PD-L1 expression was observed in 35% by TCIC, 31% by IC, and 16% by TC, with a concordance of 76%. Associations between PD-L1 and clinicopathologic factors were investigated according to receptor groups and whether the patients had received neoadjuvant chemotherapy. The three scoring methods showed differences in their associations with clinicopathologic factors in all subgroups studied. Positive PD-L1 expression by IC was significantly associated with worse overall survival in patients with neoadjuvant chemotherapy and showed a trend for worse overall survival and distant metastasis-free survival in triple-negative patients with neoadjuvant chemotherapy. Positive PD-L1 expression by TCIC and TC also showed trends for worse survival in different subgroups.

**Conclusions:**

Our findings indicate that the three scoring methods with a 1% cutoff are different in their sensitivity for PD-L1 expression and their associations with clinicopathologic factors. Scoring by TCIC is the most sensitive way to identify PD-L1-positive breast cancer by immunohistochemistry. As a prognostic marker, our study suggests that PD-L1 is associated with worse clinical outcome, most often shown by the IC score; however, the other scores may also have clinical implications in some subgroups. Large clinical trials are needed to test the similarities and differences of these scoring methods for their predictive values in anti-PD-1/PD-L1 therapy.

## Background

Over the past decade, monoclonal antibody-based immune checkpoint inhibitors targeting programmed death-1 (PD-1) and its ligand, programmed death-ligand 1 (PD-L1), have been developed and approved by the U.S. Food and Drug Administration (FDA) for the treatment of solid tumors such as non-small cell lung cancer, melanoma, urothelial carcinoma, and head and neck squamous cell carcinoma [1–5]. The FDA has also approved several diagnostic immunohistochemistry (IHC) assays corresponding to these drugs to detect PD-L1 expression and inform the selection of patients for treatment [6–11]. However, when these different immune checkpoint inhibitors are used for the same tumor type, the corresponding IHC assays may be scored differently. Taking urothelial carcinoma as an example, when the 22C3 Dako PharmDx IHC assay is used, a Combined Positive Score, which factors in expression in both tumor cells and tumor-infiltrating immune cells, is calculated, and a score of ≥ 10 is considered positive. On the other hand, the 28-8 Dako PharmDx IHC assay scores the expression in the urothelial tumor cells only, with ≥ 1% as the cutoff for positivity. The Ventana SP142 assay, in contrast, measures PD-L1 expression in the tumor-infiltrating immune cells only, with ≥ 5% staining considered positive.

The scoring of PD-L1 expression also varies in different tumor types when the same assay is used. For example, with the 22C3 Dako PharmDx IHC assay, expression in ≥ 1% of tumor cells is considered positive for non-small cell lung cancer, a Combined Positive Score of ≥ 10 is considered positive for urothelial carcinoma, and a Combined Positive Score of ≥ 1 is considered positive for gastric adenocarcinoma and cervical cancer. Overall, there is a lack of uniform guidelines in the evaluation of PD-L1 that account for different IHC assays, different cell types, and different cutoff values across tumor types.

While breast cancer is not a robustly immunogenic tumor type overall, certain subtypes, largely the estrogen receptor-negative tumors, have more abundant immune cell infiltration, representing opportunities for immune checkpoint inhibitors. Emerging clinical trials testing the utility of PD-1/PD-L1 inhibitors have brought promise to the treatment of triple-negative breast cancer (TNBC) patients [12–14]. In the phase Ib KEYNOTE-012 trial, pembrolizumab, a PD-1 inhibitor, had an overall response rate of 18.5% in patients with PD-L1-positive advanced TNBC as a single agent, and the response appeared durable [12]. In cohort B of the phase II KEYNOTE-086 study, pembrolizumab monotherapy showed durable antitumor activity as first line therapy for patients with PD-L1-positive metastatic TNBC, with an objective response rate of 21.4% [13]. In the IMpassion130 phase III trial, the PD-L1 inhibitor atezolizumab in combination with the chemotherapy drug nab-paclitaxel prolonged progression-free survival in patients with metastatic TNBC, and the survival benefit was significantly higher in PD-L1-positive TNBC than PD-L1-negative patients [14]. The last data led to the accelerated FDA approval of atezolizumab plus chemotherapy in the treatment of patients with PD-L1-positive, unresectable, locally advanced or metastatic TNBC. Trials for other subtypes of breast cancer are underway [15].

The issues experienced in the assessment of PD-L1 expression in other solid tumors are also encountered with breast cancer. Earlier studies have used various commercial antibodies to detect PD-L1 prior to FDA approval of PD-L1 IHC diagnostics [16]. More recent PD-L1 expression studies using FDA-approved antibodies have applied various scoring systems [16–23]. In the clinical trials for PD-1/PD-L1 inhibitors in TNBC, positivity was defined as PD-L1 level in the stroma or ≥ 1% of tumor cells in the KEYNOTE-012 trial (a 22C3 clone was used before the 22C3 Dako PharmDx IHC assay was available), a Combined Positive Score of ≥ 1 with the FDA-approved 22C3 Dako PharmDx IHC assay in the KEYNOTE-086 trial, and an immune cell score of ≥ 1% with the Ventana SP142 antibody in the IMpassion130 trial [12–14]. Before a uniform PD-L1 detection system is agreed upon, an understanding of the staining frequencies of different PD-L1 antibodies and the differences between scoring methods and their clinicopathologic correlates is needed for meaningful comparison of data from clinical studies and for selection of patients for anti-PD-1/PD-L1 treatment. In this study, we use the 22C3 Dako PharmDx assay, which is one of the first FDA-approved assays and widely used in many clinical laboratories, to evaluate three scoring methods for PD-L1 expression frequency and their associations with clinicopathologic factors, including stromal tumor infiltrating lymphocyte (TIL) levels, in breast cancer. The three scoring methods included a score for PD-L1 expression in the tumor cells, which is the standard approach to evaluate an IHC marker in tumor and also used in the evaluation of PD-L1 in other solid tumors such as lung cancer; a score for PD-L1 expression in tumor-infiltrating immune cells, which was used in the Impassion130 trial; and a combined tumor cell and immune cell score, which is equivalent to the method used in the KEYNOTE-086 trial. This is the first comprehensive evaluation of different scoring methods of PD-L1 in breast cancer.

## Methods

### Human breast tumor samples

This retrospective study was approved by the institutional review board of The University of Texas MD Anderson Cancer Center. Four hundred ninety-six patients diagnosed with invasive breast cancer during 2004 to 2016 and treated at our institution were included. At the last follow-up, none of the patients received anti-PD1/PD-L1 treatment as recorded in our clinical database. All samples were from surgical excision specimens. In patients with more than one tumor focus, only the largest tumor was included. Patient age, race/ethnicity, tumor size, histologic type, histologic grade, lymph node status, distant metastasis, pathologic stage, prognostic and predictive marker status, history of neoadjuvant chemotherapy (NACT), residual cancer burden category, and clinical follow-up data were retrospectively collected from slide review and patients’ medical records. Because histologic type and grade could be altered by NACT, these parameters were recorded according to the information in the pretreatment biopsy report, if the patient received NACT. The American Society of Clinical Oncology (ASCO)/College of American Pathologists (CAP) guideline recommendations [24–26] were used as references for categorizing estrogen receptor (ER), progesterone receptor (PR), and HER2 status as part of the routine pathologic evaluation. As minor modifications to the guideline for ER and PR, positive staining was defined as nuclear staining in at least 5% of invasive carcinoma, because low expression of ER and PR is clinically managed similar to ER/PR negative tumors. In the current study, patients were categorized as follows based on receptor status: positive for ER and PR but negative for HER2 (ER/PR positive group); HER2 positive, regardless of ER and PR status (HER2 group); and negative for ER, PR and HER2, or triple-negative (TNBC group).

### IHC for assessment of PD-L1 and stromal TIL

Tissue microarrays (TMAs) were constructed from representative archival paraffin blocks in the Pathology files of primary tumors using a 1.0-mm manual tissue arrayer (Beecher Instruments, Inc., Sun Prairie, WI). All blocks were from surgical excision specimens. Duplicate punches from different areas of the same tumor were obtained in 95% of the samples. Unstained tissue sections 4-μm thick were prepared from the TMAs, and IHC for PD-L1 was performed using the FDA-approved PD-L1 IHC 22C3 pharmDx kit (Dako North America Inc., Carpinteria, CA) on the Dako AutostainerLink 48 according to the manufacturer’s instructions. Slides were counterstained with Mayer’s hematoxylin. Results were evaluated with known positive and negative tissue controls. Percent PD-L1 expression in invasive tumor cells (TC) was calculated as the number of viable invasive carcinoma cells showing membranous staining of any intensity divided by the total number of viable invasive carcinoma cells. Percent PD-L1 expression in tumor-infiltrating immune cells (IC) was assessed as the proportion of tumor area occupied by PD-L1-positive immune cells of any intensity in any cell compartment. Percent PD-L1 expression in tumor-infiltrating immune cells and invasive tumor cells (TCIC) was calculated as the number of those cells showing PD-L1 staining (membranous staining for invasive tumor cells and any staining for immune cells) divided by the total number of invasive tumor cells. For each of these percentages, 1% or greater was considered positive. Of note, the TCIC percentage used in our study was equivalent to the Combined Positive Score in the KEYNOTE-086 trial [13]. For example, a TCIC of 5% was equivalent to a Combined Positive Score of 5.

On the whole slide sections from which the TMAs were generated, stromal TILs (sTILs) were evaluated as the area of the tumor stroma occupied by mononuclear inflammatory cells divided by the total tumor stromal area according to the International TILs Working Group guidelines [27, 28]. Although sTIL evaluation for the current study was conducted prior to the publication of recommendations for post-NACT TILs by the Group on breast cancer [29], the same principles were applied in this study, including assessment of sTIL within the borders of the residual tumor bed as defined by the Residual Cancer Burden [30]. For correlative analyses, ≥ 5%, ≥ 10%, and ≥ 20% were first used as cutoffs for sTILs in the current study, and associations between sTILs and clinicopathologic factors were found similar between these three cutoffs; therefore, data using only the 10% cutoff are presented below.

PD-L1 expression was evaluated by three breast pathologists HG, QD, and LH. STILs were evaluated by HG and LH. Difficult and discrepant cases were determined by discussing and reviewing at multi-headed microscopes by at least two pathologists.

### Statistical analysis

Statistical analysis was carried out using SAS 9.3 for Windows (SAS Institute Inc.) and SPSS Statistics 23.0 (IBM). Associations of PD-L1 staining and sTIL levels with clinicopathologic factors were assessed using the Fisher exact test. Multivariate analysis was performed using logistic regression or exact logistic regression, depending on the sample size, and included all clinicopathologic factors with a *p* value of 0.05 or less from the Fisher exact tests. Factors with a *p* value of 0.05 or less in the multivariate model were presented in this article. Overall survival was defined as the time from the initial breast cancer diagnosis until death from any cause or date of last follow-up. Distant metastasis-free survival was calculated as the duration between the initial breast cancer diagnosis and the time of distant metastasis. Recurrence-free survival was calculated as the duration between the initial breast cancer diagnosis and the time of either local regional recurrence or distant metastasis. Survival endpoints were estimated and plotted using the Kaplan-Meier method. Survival was compared between patient groups categorized by PD-L1 status and sTIL levels using the log-rank test. All tests were two-sided, and *p* values of 0.05 or less were considered statistically significant. For survival analysis, any *p* value between 0.05 and 0.08 was considered a trend.

## Results

### Comparison of the three PD-L1 scoring methods

**Fig. 1:**
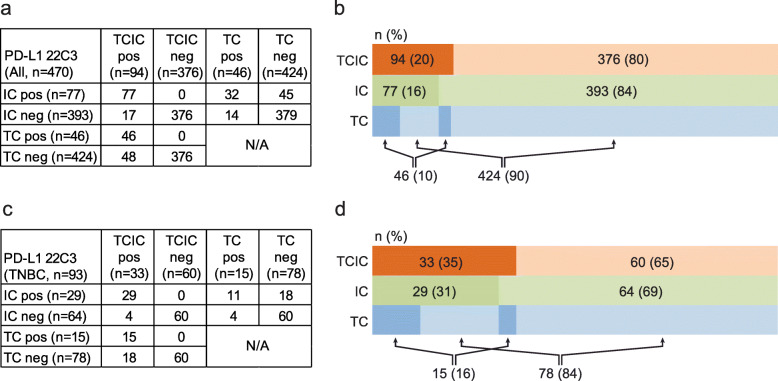
Comparison of three scoring methods in the entire cohort and in the triple-negative breast cancer patients. **a**, **b** Summary of staining results by invasive tumor cells and tumor-infiltrating immune cells (TCIC), tumor-infiltrating immune cells only (IC), and invasive tumor cells (TC) in the entire cohort. **c**, **d** Summary of staining results by TCIC, IC, and TC in the triple-negative breast cancer patients. The darker color on the left of each row of **b** and **d** represents positive cases and the lighter color on the right negative cases

**Fig. 2:**
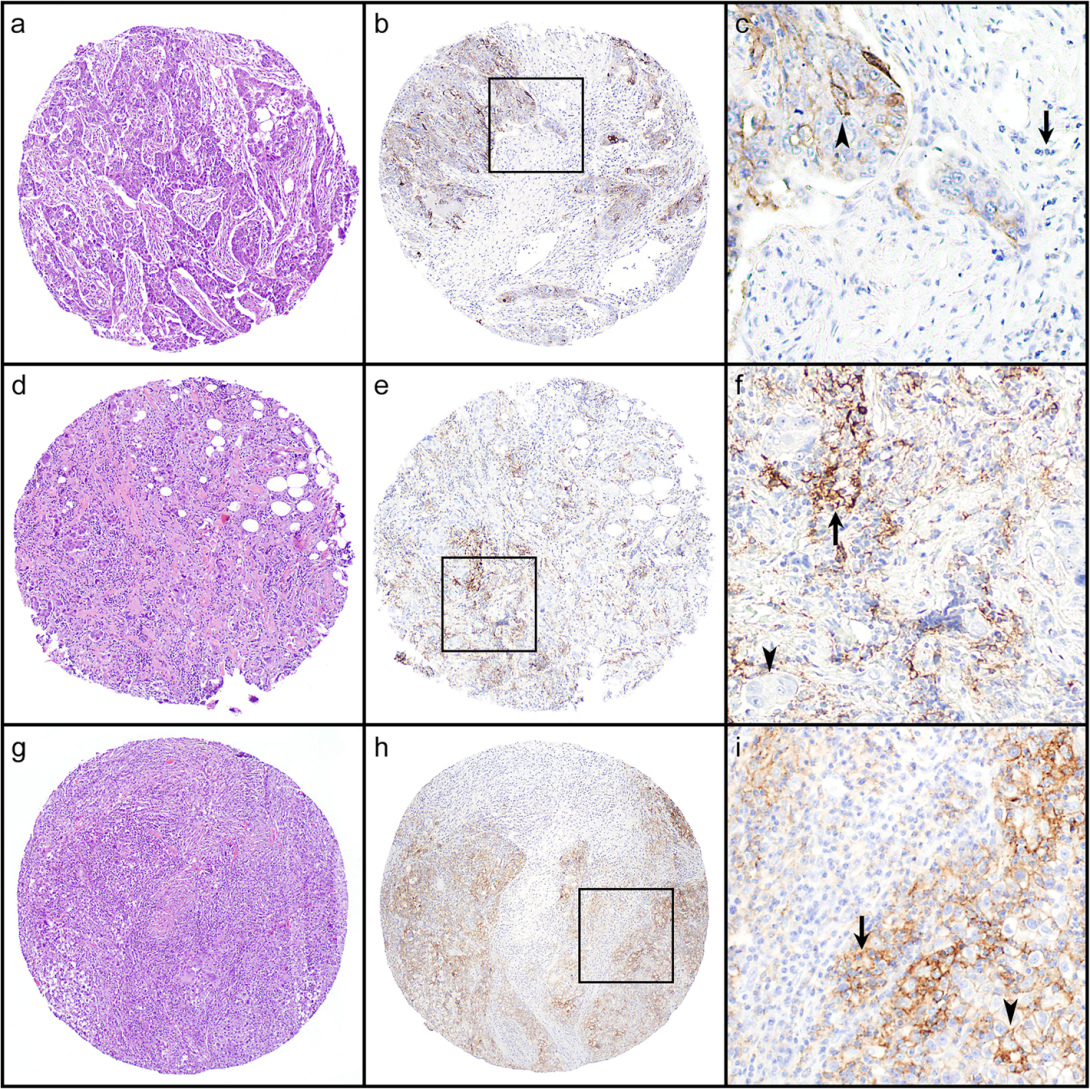
Examples of immunohistochemical staining results. **a**, **d**, **g** Hematoxylin and eosin images of the tissue microarray cores. **b**, **e**, **h** Images of PD-L1 staining. **c**, **f**, **i** Magnified images of the boxed areas in **b**, **e**, and **h**. Arrow, tumor-infiltrating immune cells. Arrowhead, tumor cells. The first row (**a**–**c**) represents a case with positive staining in the tumor cells and negative staining in the immune cells. The second row (**d**–**f**) represents a case with positive staining in the immune cells and negative staining in the tumor cells. The third row (**g**–**i**) represents a case with positive staining in both the immune cells and the tumor cells. Original magnification: **a**, **b**, **d**, **e**, **g**, **h** × 40; **c**, **f**, **i** × 100

Among the 496 patients, TCIC, TC, and IC scores for the primary breast tumors were able to be assessed in 470 patients for comparison. In the entire cohort, positive PD-L1 expression was observed in 20% of patients by TCIC, 16% by IC, and 10% by TC (Fig. 1a, b). Pair-wise comparison showed that in 87% (408/470) of patients, the staining results (positive or negative) were concordant between all scoring methods, including 7% that were positive and 80% that were negative for PD-L1. In the TNBC group (*n* = 93), positive PD-L1 expression was observed in 35% of patients by TCIC, 31% by IC, and 16% by TC (Fig. 1c, d). Concordance (positive or negative) between the three scoring methods was reached in 76% (71/93) of patients, including 11.8% that were positive and 64.5% that were negative for PD-L1. The discordance was due largely to differences between TC and the other two methods; concordance (positive or negative) between TCIC and IC was 96% in both the entire cohort and the TNBC group. Representative images of the staining results are shown in Fig. 2.

### Association between PD-L1 staining and clinicopathological factors

Among the 496 patients included in the study, 349 patients had no NACT, and 147 patients had NACT at the time of surgical excision. The associations between PD-L1 expression and clinicopathologic factors in the subgroups of patients without NACT and with NACT are summarized in Tables 1 and 2. In the subgroup without NACT, histologic grade (grade 3), sTIL level (≥ 10%), and PR status (negative) were significantly associated with positive PD-L1 staining by all the three scoring methods, while race/ethnicity (black), ER status (negative), HER2 status (positive), TNBC status (yes), and receptor group (not ER/PR positive) were significantly associated with positive PD-L1 by one or two scoring methods. In the subgroup with NACT, histologic grade (grade 3), sTIL status (≥ 10%), ER status (negative), PR status (negative), TNBC status (yes), and receptor group (not ER/PR positive) were significantly associated with positive PD-L1 staining by all scoring methods, while race/ethnicity (black) was associated with positive PD-L1 only by TC.

**Table 1:** Association of PD-L1 staining with clinicopathologic factors in all patients without neoadjuvant chemotherapy

	TC			TCIC			IC			sTIL level		
Factors	Negative	Positive	Fisher	Negative	Positive	Fisher	Negative	Positive	Fisher	Low	High	Fisher
	***N*** (%)	***N*** (%)	***P*** value	***N*** (%)	***N*** (%)	***P*** value	***N*** (%)	***N*** (%)	***P*** value	***N*** (%)	***N*** (%)	***P*** value
Age	[*N* = 341]			[*N* = 340]			[*N* = 340]			[*N* = 345]		
< 50 years	81 (88)	11 (12)	0.5411	68 (75)	23 (25)	0.2256	71 (78)	20 (22)	0.1466	60 (63)	35 (37)	**0.0153**
≥ 50 years	226 (91)	23 (9)		202 (81)	47 (19)		211 (85)	38 (15)		191 (76)	59 (24)	
Race/ethnicity	[*N* = 341]			[*N* = 340]			[*N* = 340]			[*N* = 345]		
Black	34 (83)	7 (17)	0.1159	20 (49)	21 (51)	**< 0.0001**	21 (51)	20 (49)	**< 0.0001**	17 (41)	24 (59)	**< 0.0001**
White+Latino	257 (91)	24 (9)		235 (84)	45 (16)		246 (88)	34 (12)		219 (77)	66 (23)	
Others	16 (84)	3 (16)		15 (79)	4 (21)		15 (79)	4 (21)		15 (79)	4 (21)	
Histologic type	[*N* = 340]			[*N* = 339]			[*N* = 339]			[*N* = 344]		
IDC	261 (90)	29 (10)	1	225 (78)	64 (22)	0.262	233 (81)	56 (19)	**0.0121**	200 (68)	92 (32)	**< 0.0001**
ILC	34 (89)	4 (11)		34 (89)	4 (11)		37 (97)	1 (3)		36 (95)	2 (5)	
Metaplastic	0 (0)	0 (0)		0 (0)	0 (0)		0 (0)	0 (0)		0 (0)	0 (0)	
Mixed IDC/ILC	11 (92)	1 (8)		10 (83)	2 (17)		11 (92)	1 (8)		14 (100)	0 (0)	
Histologic grade	[*N* = 340]			[*N* = 339]			[*N* = 339]			[*N* = 344]		
1 + 2	216 (96)	10 (4)	**< 0.0001**	200 (88)	26 (12)	**< 0.0001**	208 (92)	18(8)	**< 0.0001**	201 (87)	30 (13)	**< 0.0001**
3	90 (79)	24 (21)		69 (61)	44 (39)		73 (65)	40 (35)		49 (43)	64 (57)	
sTIL level	[*N* = 337]			[*N* = 337]			[*N* = 337]			NA		
< 10%	233 (95)	12 (5)	**< 0.0001**	226 (92)	19 (8)	**< 0.0001**	235 (96)	10 (4)	**< 0.0001**			
≥ 10%	70 (76)	22 (24)		42 (46)	50 (54)		44 (48)	48 (52)				
ER	[*N* = 341]			[*N* = 340]			[*N* = 340]			[*N* = 345]		
Negative	60 (86)	10 (14)	0.1828	46 (66)	24 (34)	**0.0026**	48 (69)	22 (31)	**0.0011**	27 (39)	43 (61)	**< 0.0001**
Positive	247 (91)	24 (9)		224 (83)	46 (17)		234 (87)	36 (13)		224 (81)	51 (19)	
PR	[*N* = 341]			[*N* = 340]			[*N* = 340]			[*N* = 342]		
Negative	95 (84)	18 (16)	**0.0125**	73 (65)	40 (35)	**< 0.0001**	78 (69)	35 (31)	**< 0.0001**	54 (48)	59 (52)	**< 0.0001**
Positive	212 (93)	16 (7)		197 (87)	30 (13)		204 (90)	23 (10)		197 (85)	35 (15)	
HER2	[*N* = 338]			[*N* = 337]			[*N* = 337]			[*N* = 342]		
Negative	278 (91)	29 (9)	0.2195	248 (81)	58 (19)	**0.0178**	257 (84)	49 (16)	0.0801	231 (75)	78 (25)	**0.0071**
Positive	26 (84)	5 (16)		19 (61)	12 (39)		22 (71)	9 (29)		17 (52)	16 (48)	
TNBC	[*N* = 338]			[*N* = 337]			[*N* = 337]			[*N* = 342]		
No	250 (90)	28 (10)	1	226 (81)	52 (19)	0.0518	238 (86)	40 (14)	**0.0069**	227 (80)	56 (20)	**< 0.0001**
Yes	54 (90)	6 (10)		41 (69)	18 (31)		41 (69)	18 (31)		21 (36)	38 (64)	
Receptor group	[*N* = 338]			[*N* = 337]			[*N* = 337]			[*N* = 342]		
ER/PR pos	224 (91)	23 (9)	0.4491	207 (84)	40 (16)	**0.0022**	216 (87)	31 (13)	**0.0009**	210 (84)	40 (16)	**< 0.0001**
HER2	26 (84)	5 (16)		19 (61)	12 (39)		22 (71)	9 (29)		17 (52)	16 (48)	
TNBC	54 (90)	6 (10)		41 (69)	18 (31)		41 (69)	18 (31)		21 (36)	38 (64)	
Tumor size	[*N* = 340]			[*N* = 339]			[*N* = 339]			[*N* = 344]		
≤ 2 cm	176 (92)	16 (8)	0.34	157 (81)	36 (19)	0.3325	162 (84)	31 (16)	0.5449	151 (77)	46 (23)	0.0517
> 2 to 5 cm	105 (87)	16 (13)		90 (75)	30 (25)		96 (80)	24 (20)		79 (65)	43 (35)	
> 5 cm	25 (93)	2 (7)		22 (85)	4 (15)		23 (88)	3 (12)		20 (80)	5 (20)	
pN	[*N* = 338]			[*N* = 337]			[*N* = 337]			[*N* = 342]		
(y)pN0	207 (90)	22 (10)	0.4972	185 (80)	45 (20)	0.5121	191 (83)	39 (17)	0.882	169 (73)	62 (27)	0.9442
(y)pN1	81 (91)	8 (9)		70 (80)	18 (20)		73 (83)	15 (17)		65 (71)	26 (29)	
(y)pN2	10 (91)	1 (9)		8 (73)	3 (27)		9 (82)	2 (18)		8 (67)	4 (33)	
(y)pN3	7 (78)	2 (22)		5 (63)	3 (38)		6(75)	2 (25)		6 (75)	2 (25)	
pM	[*N* = 338]			[*N* = 337]			[*N* = 337]			[*N* = 342]		
0	301 (90)	32 (10)	0.4036	265 (80)	68 (20)	1	275 (83)	58 (17)	1	245 (72)	93 (28)	1
1	4 (80)	1 (20)		3 (75)	1 (25)		4 (100)	0 (0)		3 (75)	1 (25)	
pStage	[*N* = 336]			[*N* = 335]			[*N* = 335]			[*N* = 340]		
I	163 (92)	15 (8)	0.2829	150 (84)	29 (16)	0.1412	156 (87)	23 (13)	0.1034	142 (78)	40 (22)	0.0637
II	111 (90)	13 (10)		92 (75)	31 (25)		95 (77)	28 (23)		80 (65)	44 (35)	
III	24 (83)	5 (17)		21 (72)	8 (28)		23 (79)	6 (21)		22 (73)	8 (27)	
IV	4 (80)	1 (20)		3 (75)	1 (25)		4 (100)	0 (0)		3 (75)	1 (25)	

*IDC* invasive ductal carcinoma, *ILC* invasive lobular carcinoma, *sTIL* stromal tumor infiltrating lymphocytes, *ER* estrogen receptor, *PR* progesterone receptor, *pos* positive, *TNBC* triple-negative breast cancer

**Table 2:** Association of PD-L1 staining with clinicopathologic factors in all patients with neoadjuvant chemotherapy

	TC			TCIC			IC			sTIL level		
Factors	Negative	Positive	Fisher	Negative	Positive	Fisher	Negative	Positive	Fisher	Low	High	Fisher
	***N*** (%)	***N*** (%)	***P*** value	***N*** (%)	***N*** (%)	***P*** value	***N*** (%)	***N*** (%)	***P*** value	***N*** (%)	***N*** (%)	***P*** value
Age	[*N* = 141]			[*N* = 131]			[*N* = 131]			[*N* = 145]		
< 50 years	50 (94)	3 (6)	0.535	45 (88)	6 (12)	0.1651	47 (92)	4 (8)	0.1257	41 (73)	15 (27)	0.557
≥ 50 years	79 (90)	9 (10)		62 (78)	18 (23)		65 (81)	15 (19)		69 (78)	20 (22)	
Race/ethnicity	[*N* = 141]			[*N* = 131]			[*N* = 131]			[*N* = 145]		
Black	19 (79)	5 (21)	**0.0228**	18 (75)	6 (25)	0.5126	20 (83)	4 (17)	0.9225	16 (70)	7 (30)	0.6991
White+Latino	97 (95)	5 (5)		78 (84)	15 (16)		80 (86)	13 (14)		82 (77)	24 (23)	
Others	13 (87)	2 (13)		11 (79)	3 (21)		12 (86)	2 (14)		12 (75)	4 (25)	
Histologic type	[*N* = 141]			[*N* = 131]			[*N* = 131]			[*N* = 145]		
IDC	106 (91)	10 (9)	0.0737	85 (79)	22 (21)	0.0624	89 (83)	18 (17)	0.2508	87 (73)	33 (28)	0.1731
ILC	17 (100)	0 (0)		16 (100)	0 (0)		16 (100)	0 (0)		16 (94)	1 (6)	
Metaplastic	3 (60)	2 (40)		3 (60)	2 (40)		4 (80)	1 (20)		4 (80)	1 (20)	
Mixed IDC/ILC	3 (100)	0 (0)		3 (100)	0 (0)		3 (100)	0 (0)		3 (100)	0 (0)	
Histologic grade^a^	[*N* = 141]			[*N* = 131]			[*N* = 131]			[*N* = 145]		
1 + 2	68 (100)	0 (0)	**0.0003**	62 (95)	3 (5)	**0.0001**	62 (95)	3 (5)	**0.0022**	65 (88)	9 (12)	**0.0008**
3	61 (84)	12 (16)		45 (68)	21 (32)		50 (76)	16 (24)		45 (63)	26 (37)	
sTIL level	[*N* = 139]			[*N* = 131]			[*N* = 131]			NA		
< 10%	102 (97)	3 (3)	**0.0002**	89 (91)	9 (9)	**< 0.0001**	90 (92)	8 (8)	**0.0006**			
≥ 10%	25 (74)	9 (26)		16 (52)	15 (48)		20 (65)	11 (35)				
ER	[*N* = 141]			[*N* = 131]			[*N* = 131]			[*N* = 145]		
Negative	30 (77)	9 (23)	**0.0005**	20 (57)	15 (43)	**< 0.0001**	24 (69)	11 (31)	**0.0019**	22 (56)	17 (44)	**0.0018**
Positive	99 (97)	3 (3)		87 (91)	9 (9)		88 (92)	8 (8)		88 (83)	18 (17)	
PR	[*N* = 141]			[*N* = 131]			[*N* = 131]			[*N* = 145]		
Negative	48 (83)	10 (17)	**0.0037**	34 (67)	17 (33)	**0.0009**	39 (76)	12 (24)	**0.0236**	36 (62)	22 (38)	**0.0026**
Positive	81 (98)	2 (2)		73 (91)	7 (9)		73 (91)	7 (9)		74 (85)	13 (15)	
HER2	[*N* = 141]			[*N* = 131]			[*N* = 131]			[*N* = 145]		
Negative	118 (91)	12 (9)	0.5987	98(81)	23 (19)	0.6881	103 (85)	18 (15)	1	99 (75)	33 (25)	0.7344
Positive	11 (100)	0 (0)		9(90)	1 (10)		9 (90)	1 (10)		11 (85)	2 (15)	
TNBC	[*N* = 141]			[*N* = 131]			[*N* = 131]			[*N* = 145]		
No	101 (97)	3 (3)	**0.0003**	88 (91)	9 (9)	**< 0.0001**	89 (92)	8 (8)	**0.0014**	90 (83)	18 (17)	**0.0007**
Yes	28 (76)	9 (24)		19 (56)	15 (44)		23 (68)	11 (32)		20 (54)	17 (46)	
Receptor group	[*N* = 141]			[*N* = 131]			[*N* = 131]			[*N* = 145]		
ER/PR pos	90 (97)	3 (3)	**0.0012**	79 (91)	8 (9)	**0.0001**	80 (92)	7 (8)	**0.0038**	79 (83)	16 (17)	**0.0023**
HER2	11 (100)	0 (0)		9 (90)	1 (10)		9 (90)	1 (10)		11 (85)	2 (15)	
TNBC	28 (76)	9 (24)		19 (56)	15 (44)		23 (68)	11 (32)		20 (54)	17 (46)	
Tumor size	[*N* = 141]			[*N* = 131]			[*N* = 131]			[*N* = 145]		
≤ 2 cm	26 (84)	5 (16)	0.2433	19 (73)	7 (27)	0.4286	20 (77)	6 (23)	0.3765	21 (62)	13 (38)	**0.049**
> 2 to 5 cm	54 (93)	4 (7)		49 (84)	9 (16)		51 (88)	7 (12)		43 (75)	14 (25)	
> 5 cm	49 (94)	3 (6)		39 (83)	8 (17)		41 (87)	6 (13)		46 (85)	8 (15)	
ypN	[*N* = 141]			[*N* = 131]			[*N* = 131]			[*N* = 145]		
(y)pN0	39 (85)	7 (15)	0.0921	33 (77)	10 (23)	0.6247	36 (84)	7 (16)	0.9424	35 (74)	12 (26)	0.3048
(y)pN1	45 (96)	2 (4)		36 (84)	7 (16)		36 (84)	7 (16)		37 (74)	13 (26)	
(y)pN2	22 (88)	3 (12)		19 (79)	5 (21)		21 (88)	3 (13)		18 (69)	8 (31)	
(y)pN3	23 (100)	0 (0)		19 (90)	2 (10)		19 (90)	2 (10)		20 (91)	2 (9)	
pM	[*N* = 141]			[*N* = 131]			[*N* = 131]			[*N* = 145]		
0	119 (91)	12 (9)	1	101 (82)	22 (18)	0.6382	106 (86)	17 (14)	0.3272	100 (74)	35 (26)	0.1184
1	10 (100)	0 (0)		6 (75)	2 (25)		6 (75)	2 (25)		10 (100)	0 (0)	
RCB category	[*N* = 140]			[*N* = 130]			[*N* = 130]			[*N* = 144]		
I	4 (80)	1 (20)	0.5155	3 (75)	1 (25)	0.4125	3 (75)	1 (25)	0.2473	5 (83)	1 (17)	0.9425
II	63 (91)	6 (9)		54 (86)	9 (14)		57 (90)	6 (10)		56 (77)	17 (23)	
III	61 (92)	5 (8)		49 (78)	14 (22)		51 (81)	12 (19)		48 (74)	17 (26)	

^a^Histologic grade for post-treatment tumors was based on pre-treatment grade

*IDC* invasive ductal carcinoma, *ILC* invasive lobular carcinoma, *sTIL* stromal tumor infiltrating lymphocytes, *ER* estrogen receptor, *PR* progesterone receptor, *TNBC* triple-negative breast cancer, *pos* positive, *RCB* residual cancer burden

With regard to receptor status, the entire cohort included 348 patients in the ER/PR positive group, 46 patients in the HER2 group, and 99 patients in the TNBC group. The results for the ER/PR positive group are shown in Additional file 1, Tables S1 and S2. STIL level (≥ 10%) was significantly associated with positive PD-L1 staining by all scoring methods in both the subgroup with NACT and the subgroup without NACT. Race/ethnicity (black) and histologic grade (grade 3) were associated with positive PD-L1 by at least one of the scoring methods.

In the HER2 group, histologic grade (grade 3), sTIL level (≥ 10%), ER status (negative), and PR status (negative) were significantly associated with positive PD-L1 staining by at least one of the scoring methods in the subgroup without NACT (Additional file 1, Table S3). There were too few patients in the HER2 subgroup with NACT for meaningful statistical analysis (Additional file 1, Table S4).

In the TNBC group, race/ethnicity (black) and sTIL level (≥ 10%) were significantly associated with positive PD-L1 staining by TCIC and IC scores in the subgroup without NACT (Table 3). In the subgroup with NACT, age (≥ 50 years) was the only factor associated with PD- L1 staining, by TCIC (Table 4).

**Table 3:** Association of PD-L1 staining with clinicopathologic factors in triple-negative patients without neoadjuvant chemotherapy

	TC			TCIC			IC			sTIL level		
Factors	Negative	Positive	Fisher	Negative	Positive	Fisher	Negative	Positive	Fisher	Low	High	Fisher
	***N*** (%)	***N*** (%)	***P*** value	***N*** (%)	***N*** (%)	***P*** value	***N*** (%)	***N*** (%)	***P*** value	***N*** (%)	***N*** (%)	***P*** value
Age	[*N* = 60]			[*N* = 59]			[*N* = 59]			[*N* = 59]		
< 50 years	13 (87)	2 (13)	0.6339	11 (79)	3 (21)	0.5163	11 (79)	3 (21)	0.5163	6 (43)	8 (57)	0.5378
≥ 50 years	41 (91)	4 (9)		30 (67)	15 (33)		30 (67)	15 (33)		15 (33)	30 (67)	
Race/ethnicity	[*N* = 60]			[*N* = 59]			[*N* = 59]			[*N* = 59]		
Black	13 (81)	3 (19)	0.3966	6 (38)	10 (63)	**0.003**	6 (38)	10 (63)	**0.003**	3 (19)	13 (81)	0.1211
White+Latino	40 (93)	3 (7)		34 (81)	8 (19)		34 (81)	8 (19)		17 (40)	25 (60)	
Others	1 (100)	0 (0)		1 (100)	0 (0)		1 (100)	0 (0)		1 (100)	0 (0)	
Histologic type	[*N* = 59]			[*N* = 58]			[*N* = 58]			[*N* = 58]		
IDC	53 (90)	6 (10)	NA	40 (69)	18 (31)	NA	40 (69)	18 (31)	NA	20 (34)	38 (66)	NA
Metaplastic	0 (0)	0 (0)		0 (0)	0 (0)		0 (0)	0 (0)		0 (0)	0 (0)	
Histologic grade	[*N* = 59]			[*N* = 58]			[*N* = 58]			[*N* = 58]		
2	11 (100)	0 (0)	0.582	8 (73)	3 (27)	1	8 (73)	3 (27)	1	5 (45)	6 (55)	0.4866
3	42 (88)	6 (13)		32 (68)	15 (32)		32 (68)	15 (32)		15 (32)	32 (68)	
sTIL level	[*N* = 59]			[*N* = 59]			[*N* = 59]			NA		
< 10%	21 (100)	0 (0)	0.0796	21 (100)	0 (0)	**0.0001**	21 (100)	0 (0)	**0.0001**			
≥ 10%	32 (84)	6 (16)		20 (53)	18 (47)		20 (53)	18 (47)				
Tumor size	[*N* = 59]			[*N* = 58]			[*N* = 58]			[*N* = 58]		
≤ 2 cm	31 (97)	1 (3)	0.14	25 (78)	7 (22)	0.1144	25 (78)	7 (22)	0.1144	14 (44)	18 (56)	0.2326
> 2 to 5 cm	19 (83)	4 (17)		14 (61)	9 (39)		14 (61)	9 (39)		6 (26)	17 (74)	
> 5 cm	3 (75)	1 (25)		1 (33)	2 (67)		1 (33)	2 (67)		0 (0)	3 (100)	
pN	[*N* = 59]			[*N* = 58]			[*N* = 58]			[*N* = 58]		
(y)pN0	37 (90)	4 (10)	0.7838	30 (73)	11 (27)	0.559	30 (73)	11 (27)	0.559	15 (37)	26 (63)	0.4834
(y)pN1	11 (85)	2 (15)		7 (54)	6 (46)		7 (54)	6 (46)		3 (23)	10 (77)	
(y)pN2	3 (100)	0 (0)		2 (67)	1 (33)		2 (67)	1 (33)		1 (33)	2 (67)	
(y)pN3	2 (100)	0 (0)		1 (100)	0 (0)		1 (100)	0 (0)		1 (100)	0 (0)	
pM	[*N* = 59]			[*N* = 58]			[*N* = 58]			[*N* = 58]		
0	51 (89)	6 (11)	1	39 (68)	18 (32)	1	39 (68)	18 (32)	1	20 (35)	37 (65)	1
1	2 (100)	0 (0)		1 (100)	0 (0)		1 (100)	0 (0)		0 (0)	1 (100)	
pStage	[*N* = 57]			[*N* = 56]			[*N* = 56]			[*N* = 56]		
I	26 (93)	2 (7)	0.6264	22 (79)	6 (21)	0.3932	22 (79)	6 (21)	0.3932	12 (43)	16 (57)	0.437
II	19 (86)	3 (14)		13 (59)	9 (41)		13 (59)	9 (41)		5 (23)	17 (77)	
III	4 (80)	1 (20)		3 (60)	2 (40)		3 (60)	2 (40)		2 (40)	3 (60)	
IV	2 (100)	0 (0)		1 (100)	0 (0)		1 (100)	0 (0)		0 (0)	1 (100)	

*IDC* invasive ductal carcinoma, *sTIL* stromal tumor infiltrating lymphocytes

**Table 4:** Association of PD-L1 staining with clinicopathologic factors in triple-negative patients with neoadjuvant chemotherapy

	TC			TCIC			IC			sTIL level		
Factors	Negative	Positive	Fisher	Negative	Positive	Fisher	Negative	Positive	Fisher	Low	High	Fisher
	***N*** (%)	***N*** (%)	***P*** value	***N*** (%)	***N*** (%)	***P*** value	***N*** (%)	***N*** (%)	***P*** value	***N*** (%)	***N*** (%)	***P*** value
Age	[*N* = 37]			[*N* = 34]			[*N* = 34]			[*N* = 37]		
< 50 years	14 (93)	1 (7)	0.0557	12 (80)	3 (20)	**0.0171**	13 (87)	2 (13)	0.064	10 (63)	6 (38)	0.5085
≥ 50 years	14 (64)	8 (36)		7 (37)	12 (63)		10 (53)	9 (47)		10 (48)	11 (52)	
Race/ethnicity	[*N* = 37]			[*N* = 34]			[*N* = 34]			[*N* = 37]		
Black	2 (40)	3 (60)	0.1176	2 (40)	3 (60)	0.6382	3 (60)	2 (40)	0.4917	1 (25)	3 (75)	0.6123
White+Latino	23 (82)	5 (18)		14 (56)	11 (44)		16 (64)	9 (36)		17 (59)	12 (41)	
Others	3 (75)	1 (25)		3 (75)	1 (25)		4 (100)	0 (0)		2 (50)	2 (50)	
Histologic type	[*N* = 37]			[*N* = 34]			[*N* = 34]			[*N* = 37]		
IDC	26 (79)	7 (21)	0.2436	17 (57)	13 (43)	1	20 (67)	10 (33)	1	17 (52)	16 (48)	0.6088
Metaplastic	2 (50)	2 (50)		2 (50)	2 (50)		3 (75)	1 (25)		3 (75)	1 (25)	
Histologic grade^a^	[*N* = 37]			[*N* = 34]			[*N* = 34]			[*N* = 37]		
2	1 (100)	0 (0)	1	1 (100)	0 (0)	1	1 (100)	0 (0)	1	2 (67)	1 (33)	1
3	27 (75)	9 (25)		18 (55)	15 (45)		22 (67)	11 (33)		18 (53)	16 (47)	
sTIL level	[*N* = 35]			[*N* = 32]			[*N* = 32]			NA		
< 10%	16 (84)	3 (16)	0.2453	11 (61)	7 (39)	0.4765	12 (67)	6 (33)	1			
≥ 10%	10 (63)	6 (38)		6 (43)	8 (57)		9 (64)	5 (36)				
Tumor size	[*N* = 37]			[*N* = 34]			[*N* = 34]			[*N* = 37]		
≤ 2 cm	9 (69)	4 (31)	0.796	6 (60)	4 (40)	0.8287	7 (70)	3 (30)	1	6 (43)	8 (57)	0.158
> 2 to 5 cm	8 (80)	2 (20)		6 (60)	4 (40)		7 (70)	3 (30)		3 (38)	5 (63)	
> 5 cm	11 (79)	3 (21)		7 (50)	7 (50)		9 (64)	5 (36)		11 (73)	4 (27)	
ypN	[*N* = 37]			[*N* = 34]			[*N* = 34]			[*N* = 37]		
(y)pN0	14 (74)	5 (26)	0.5435	11 (65)	6 (35)	0.4114	13 (76)	4 (24)	0.6365	11 (61)	7 (39)	0.1457
(y)pN1	6 (86)	1 (14)		4 (67)	2 (33)		4 (67)	2 (33)		2 (25)	6 (75)	
(y)pN2	4 (57)	3 (43)		2 (29)	5 (71)		4 (57)	3 (43)		4 (50)	4 (50)	
(y)pN3	4 (100)	0 (0)		2 (50)	2 (50)		2 (50)	2 (50)		3 (100)	0 (0)	
pM	[*N* = 37]			[*N* = 34]			[*N* = 34]			[*N* = 37]		
0	25 (74)	9 (26)	0.5622	18 (58)	13 (42)	0.5714	22 (71)	9 (29)	0.239	17 (50)	17 (50)	0.2342
1	3 (100)	0 (0)		1 (33)	2 (67)		1 (33)	2 (67)		3 (100)	0 (0)	
RCB category	[*N* = 37]			[*N* = 34]			[*N* = 34]			[*N* = 37]		
I	1 (50)	1 (50)	0.4578	0 (0)	1 (100)	0.059	0 (0)	1 (100)	0.0824	1 (50)	1 (50)	0.8668
II	16 (80)	4 (20)		13 (72)	5 (28)		15 (83)	3 (17)		12 (57)	9 (43)	
III	11 (73)	4 (27)		6 (40)	9 (60)		8 (53)	7 (47)		7 (50)	7 (50)	

^a^Histologic grade for post-treatment tumors was based on pre-treatment grade

*IDC* invasive ductal carcinoma, *sTIL* stromal tumor infiltrating lymphocytes, *RCB* residual cancer burden

Multivariate analysis was conducted in all patients with NACT and without NACT, and the ER/PR positive subgroup without NACT. Other subgroups had relatively small numbers of patients. As shown in Tables 5 and 6 and Additional file 1, Table S5, sTIL (≥ 10%) retained a significant association with positive PD-L1 staining in all these subgroups by each scoring method. Black race/ethnicity (vs. white and Latino), histologic grade (grade 3), and TNBC group (vs. ER/PR positive group) were also significantly associated with positive PD-L1 staining by at least one of the scoring methods in the all patient subgroups.

**Table 5:** Summary of multivariate analysis in all patients without neoadjuvant chemotherapy showing the odds ratio (95% confidence interval) of variables significantly associated with PD-L1 scoring methods and sTIL level

Factor	TC	TCIC	IC	sTIL level
Age				
≥ 50 vs. < 50 years				0.50 (0.27, 0.92)^a^
Race/ethnicity				
White+Latino vs. Black		0.32 (0.14, 0.74)^b^	0.21 (0.09, 0.53)^c^	
Others vs. Black		NS	NS	
Histologic grade				
3 vs. 1 + 2	3.31 (1.38,7.93)^b^			4.69 (2.57, 8.57)^c^
sTIL level				
≥ 10% vs. < 10%	3.48 (1.50, 8.11)^b^	12.44 (6.59, 23.49)^c^	23.12 (10.60, 50.42)^c^	
Receptor group				
ER/PR pos vs. TNBC				0.20 (0.10, 0.43)^c^
HER2 vs. TNBC				NS

*NS* not significant

^a^*P* value < 0.05–0.01

^b^*P* value < 0.01–0.001

^c^*P* value < 0.001

**Table 6:** Summary of multivariate analysis in all patients with neoadjuvant chemotherapy showing the odds ratio (95% confidence interval) of variables significantly associated with PD-L1 scoring methods and sTIL level

Factor	TC22C3	TCIC22C3	IC22C3	sTIL level
Race/ethnicity				
White+Latino vs. Black	0.10 (0.01, 0.76)^a^			
Others vs. Black	NS			
Histologic grade				
3 vs. 1 + 2		7.27 (1.94, 27.24)^b^	4.94 (1.31,18.73)^a^	4.38 (1.84, 10.45)^c^
sTIL level				
≥ 10% vs. < 10%	6.10 (1.18, 41.16)^a^	6.71 (2.37, 18.99)^c^	4.39 (1.49, 12.85)^b^	
Receptor group				
ER/PR pos vs. TNBC	0.10 (0.01, 0.63)^b^			
HER2 vs. TNBC	NS			
Tumor size				
> 2 to 5 cm vs. ≤2 cm				NS
> 5 cm vs. ≤2 cm				0.26 (0.09, 0.76)^a^

*NS* not significant

^a^*P* value < 0.05–0.01

^b^*P* value < 0.01–0.001

^c^*P* value < 0.001

### Associations between sTIL, PD-L1 status, and other clinicopathologic factors

In the entire cohort comprising all receptor groups, higher sTIL level (≥ 10%) was associated with histologic grade (grade 3), ER status (negative), PR status (negative), TNBC status (yes), and receptor group (not ER/PR positive) both in the subgroup with NACT and the subgroup without NACT (Tables 1 and 2). In addition, higher sTIL level was associated with age (< 50 years), race/ethnicity (black), histologic type (invasive ductal carcinoma), and HER2 status (positive) in the subgroup without NACT and with tumor size (smaller tumor) in the subgroup with NACT. Higher sTIL level was also associated with age (< 50 years), race/ethnicity (black), histologic type (invasive ductal carcinoma), and histologic grade (grade 3) in the ER/PR positive group without NACT, and with age (< 50 years) in the HER2 group without NACT (Additional file 1, Tables S1 and S3). Interestingly, while sTIL level was significantly associated with TNBC status in the entire cohort (Tables 1 and 2), it was not associated with any clinicopathologic factors in the TNBC group (Tables 3 and 4).

Higher sTIL level was associated with positive PD-L1 staining by all three scoring methods in the entire cohort and in the ER/PR positive group with or without NACT (Tables 1 and 2, Additional file 1, Tables S1 and S2). The direct association was also seen in the HER2 and TNBC subgroups without NACT by TCIC and IC, but not by TC (Table 3 and Additional file 1, Table S3). Since both PD-L1 and sTIL level were associated with receptor status (Tables 1 and 2), the associations between sTIL, PD-L1 expression, and receptor status were further explored. As shown in Tables 7 and 8, when patients were grouped by sTIL level (≥ 10% vs. < 10%), positive PD-L1 expression by each scoring method was significantly associated with TNBC receptor group (vs. ER/PR positive group) only in the lower sTIL subgroup with NACT, suggesting that for patients without NACT or with high stromal TIL levels, receptor status does not affect PD-L1 expression.

**Table 7:** Association of PD-L1 staining with estrogen receptor/progesterone receptor-positive and triple-negative status in sTIL subgroups in patients without neoadjuvant chemotherapy

	TC				TCIC				IC			
	sTIL < 10%		sTIL ≥10%		sTIL < 10%		sTIL ≥10%		sTIL < 10%		sTIL ≥10%	
PD-L1	Neg	Pos	Neg	Pos	Neg	Pos	Neg	Pos	Neg	Pos	Neg	Pos
ER/PR pos	195	10	26	13	188	17	17	22	195	10	18	21
TNBC	21	0	32	6	21	0	20	18	21	0	20	18
Fisher *P* value	0.6044		0.1121		0.3787		0.4917		0.6044		0.6509	

*ER* estrogen receptor, *PR* progesterone receptor, *TNBC* triple-negative breast cancer, *sTIL* stromal tumor infiltrating lymphocytes, *pos* positive, *neg* negative

**Table 8:** Association of PD-L1 staining with estrogen receptor/progesterone receptor-positive and triple-negative status in sTIL subgroups in patients with neoadjuvant chemotherapy

	TC				TCIC				IC			
	sTIL < 10%		sTIL ≥10%		sTIL < 10%		sTIL ≥10%		sTIL < 10%		sTIL ≥10%	
PD-L1	Neg	Pos	Neg	Pos	Neg	Pos	Neg	Pos	Neg	Pos	Neg	Pos
ER/PR pos	77	0	13	3	70	2	9	6	70	2	10	5
TNBC	16	3	10	6	11	7	6	8	12	6	9	5
Fisher *P* value	**0.0068**		0.4331		**0.0001**		0.4661		**0.0006**		1	

*ER* estrogen receptor, *PR* progesterone receptor, *TNBC* triple-negative breast cancer, *sTIL* stromal tumor infiltrating lymphocytes, *pos* positive, *neg* negative

In the multivariate analysis, younger age (< 50 years), histologic grade (grade 3), TNBC receptor group (vs. ER/PR positive group), and a tumor size of ≤ 2 cm (vs. > 5 cm) were significantly associated with higher sTIL level in at least one of the three subgroups (all patients without NACT, all patients with NACT, and the ER/PR positive patients without NACT) tested (Tables 5 and 6 and Additional file 1, Table S5). Among these factors, histologic grade (grade 3) was the only significant factor in all of these subgroups.

### Association between race/ethnicity, PD-L1 status, sTIL, and receptor status

Race/ethnicity (black) was frequently significantly associated with positive PD-L1 staining in both univariate and multivariate analyses (Tables 1, 2, 3, 5, and 6, Additional file 1, Tables S1, S2, and S5). Additional univariate analysis showed significant association between race/ethnicity and receptor status in the subgroup without NACT, indicating that black race/ethnicity was significantly associated with TNBC status (*p* = 0.001; data not shown). STIL level was also associated with receptor group and race/ethnicity in the subgroup without NACT (Table 1). To further understand the impact of race/ethnicity on PD-L1 staining, we analyzed TCIC and IC scores, which were significantly associated with race/ethnicity in the multivariate analysis (Table 5), in the black race/ethnicity subgroup without NACT in a multivariate model including sTIL, receptor group, and histologic grade. Of these factors, only higher sTIL level (≥ 10%) was independently associated with positive PD-L1 staining (*p* = 0.0003, odds ratio 27, 95% CI 4.57–159.67, for both TCIC and IC).

### Association of PD-L1 and sTIL with prognosis

**Fig. 3:**
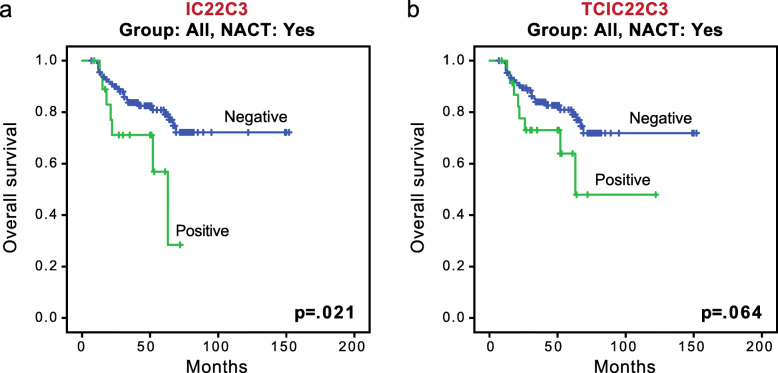
Association of PD-L1 expression with survival in the entire cohort. **a** Kaplan-Meier curves of overall survival between tumors with positive and negative PD-L1 expression by IC in the subgroup with neoadjuvant chemotherapy (NACT). **b** Kaplan-Meier curves of overall survival between tumors with positive and negative PD-L1 expression by TCIC in the subgroup with NACT

Overall survival, recurrence-free survival, and distant metastasis-free survival were evaluated according to PD-L1 expression and sTIL level for the 495 patients for whom follow-up data were available. Follow-up times ranged from 3 months to 154 months (median follow-up, 48 months). As shown in Fig. 3, in the entire cohort, positive PD-L1 staining by IC was significantly associated with worse overall survival in the subgroup with NACT (*p* = 0.021; Fig. 3a). In the same subgroup, positive PD-L1 staining by TCIC showed a trend for worse overall survival (*p* = 0.064; Fig. 3b).

**Fig. 4:**
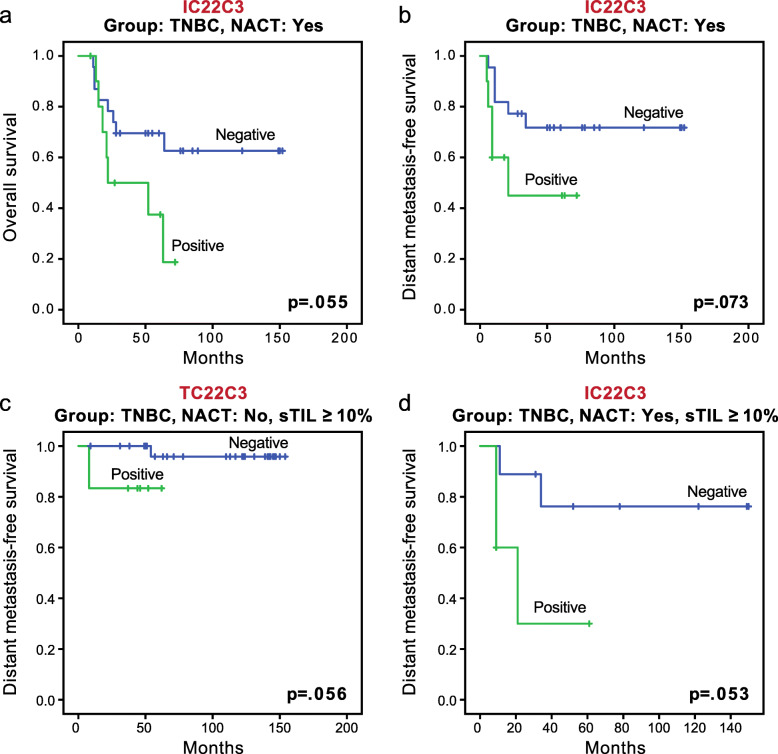
Association of PD-L1 expression with survival in triple-negative breast cancer (TNBC) patients. **a** Kaplan-Meier curves of overall survival between tumors with positive and negative PD-L1 expression by IC in the TNBC subgroup with NACT. **b** Kaplan-Meier curves of distant metastasis-free survival between tumors with positive and negative PD-L1 expression by IC in the TNBC subgroup with NACT. **c** Kaplan-Meier curves of distant metastasis-free survival between tumors with positive and negative PD-L1 expression by TC in the TNBC group without NACT and with higher stromal tumor-infiltrating lymphocyte (sTIL) level (≥ 10%). **d** Kaplan-Meier curves of distant metastasis-free survival between tumors with positive and negative PD-L1 expression by IC in the TNBC group with NACT and higher sTIL level (≥ 10%)

In the TNBC group, positive PD-L1 staining by IC showed a trend for worse overall survival (*p* = 0.055; Fig. 4a) and worse distant metastasis-free survival (*p* = 0.073; Fig. 4b) in the subgroup with NACT. In the ER/PR positive group and HER2 group, no significant association was seen between PD-L1 expression and survival.

A trend for better recurrence-free survival was observed for higher sTIL level in the TNBC group without NACT (*p* = 0.076, Additional file 1, Fig. S1). The association of PD-L1 staining with survival in sTIL subgroups was also investigated. In TNBC patients without NACT and with higher sTIL (≥ 10%), positive PD-L1 staining by TC showed a trend for worse distant metastasis-free survival (*p* = 0.056; Fig. 4c). In TNBC patients with NACT and higher sTIL, positive PD-L1 staining by IC showed a trend for worse distant metastasis-free survival (*p* = 0.053; Fig. 4d). No significant association or trend was observed between PD-L1 and survival in TNBC patients with lower sTIL levels, or in the entire cohort or other receptor subgroups when patients were grouped by sTIL level.

### PD-L1 expression using TCIC 10% cutoff and association with prognosis

While the manuscript of our study was being reviewed, a press release regarding the KEYNOTE-355 trial announced that pembrolizumab plus chemotherapy significantly improved progression-free survival compared to chemotherapy alone in patients with metastatic TNBC whose tumor expressed PD-L1 with a Combined Positive Score of ≥ 10 (https://bit.ly/2HtT4rj; unpublished data). It is possible that in the near future, this new cutoff will be applied in clinical settings. Therefore, it was of interest to examine the expression of PD-L1 in our cohort using a cutoff of ≥ 10% by TCIC, which would be equivalent to a Combined Positive Score of ≥ 10. In the entire cohort, positive PD-L1 expression was seen in 10% of patients (47/471), including 11% (36/340) of those without NACT and 8% (11/131) of those with NACT. In the TNBC group, positive PD-L1 expression was found in 19% (18/93) of patients, including 19% (11/59) of those without NACT and 21% (7/34) of those with NACT. Overall survival, recurrence-free survival, and distant metastasis-free survival were also evaluated in the entire cohort and in the TNBC patients using the TCIC ≥ 10% cutoff. No significant association or trend was observed between PD-L1 expression and prognosis in those subgroups with or without NACT (*p* > 0.08).

## Discussion

Previous studies have demonstrated that PD-L1 expression in breast cancer is positively associated with high TIL levels and with the presence of poor prognostic factors such as high histologic grade, negative ER and PR status, positive HER2 status, and TNBC status [5, 16, 31]. Similar associations were observed in our study by using different scoring methods. In the entire cohort, PD-L1 positivity by each scoring method was associated with higher nuclear grade, higher sTIL level, and negative PR status both with and without NACT in the univariate analysis; however, the scoring methods showed differences for other clinicopathologic factors. For example, in the subgroup without NACT, HER2 status was associated with only the TCIC score, and TNBC status was associated with only the IC score.

In the multivariate analysis, sTIL remained significantly associated with PD-L1 positivity by all scoring methods in the entire cohort; however, race/ethnicity was significantly associated with TCIC and IC, but not TC, in the subgroup without NACT, and histologic grade was significantly associated with TCIC and IC, but not TC, in the subgroup with NACT (Tables 5 and 6). Interestingly, even though black race/ethnicity was associated with TNBC in the entire cohort without NACT, supporting previous studies [32, 33], when PD-L1 expression by TCIC and IC in the black race/ethnicity subgroup without NACT was investigated in a multivariate model, only sTIL level, not receptor status, was independently associated with TCIC and IC, suggesting that in black patients, sTIL level is a stronger predictor than receptor status for PD-L1 expression. It may appear that TCIC and IC scores had similar associations with clinicopathologic factors across various subgroups; however, in the ER/PR positive subgroup without NACT, the same factors were significantly associated with TC and TCIC, while race/ethnicity was significantly associated with only IC in the multivariate analysis (Additional file 1, Table S5). Thus, in the same subgroup of patients, PD-L1 positivity may be associated with different clinicopathologic factors depending on the scoring method.

In our study, PD-L1 positivity by IC was significantly associated with worse overall survival in all patients with NACT, whereas TCIC showed a similar trend. In the TNBC subgroup with NACT, PD-L1 positivity by IC showed trends for worse overall survival and distant metastasis-free survival. These results are consistent with a previous report that demonstrated PD-L1 as a poor prognostic factor in post NACT residual TNBC [34]. The prognostic value of PD-L1 expression by IHC in breast cancer has conflicted between previous studies, partially owing to technical issues related to different antibody clones, cutoff points, and scoring systems. While some studies demonstrated a direct correlation between PD-L1 expression and clinical outcome, others identified PD-L1 as an indicator for worse survival, or no association was found [16, 17, 19, 20, 22, 23, 31]. Some of these studies were performed before the FDA approval of PD-L1 IHC diagnostics, indicating that these discrepancies may be attributed in part to a difference in PD-L1 detection antibodies. But even in studies using FDA-approved, commercially standardized clones, the prognostic value of PD-L1 still was not consistent (Table 9). Of note, the scoring systems in those studies varied whether tumor cells and/or immune cells were assessed, suggesting that both different clones and different scoring systems played a role in reaching the conclusions. Furthermore, although the TC scores in those studies were presumably comparable, the IC scores were often not clearly defined [19, 21]. Importantly, the IC score in the current study, which was adopted from the IMpassion130 trial [14], is the proportion of tumor area occupied by PD-L1-positive immune cells, different from the typical IHC interpretation where the denominator is the total number of cells. Also, because the TCIC score used in our study (equivalent to the Combined Positive Score in the KEYNOTE-086 trial [13]) measures the number of PD-L1 staining immune cells and invasive tumor cells divided by the total number of invasive tumor cells, the TCIC score does not represent the sum of TC and IC.

**Table 9:** Summary of recent published studies of PD-L1 expression in breast cancer using FDA-approved clones

Reference	No., type of breast tumors	Clones	Pathologic material	Cutoffs for positive/high staining	Correlation with prognosis
He et al. [16]	68, IBC, post NACT	28–8	TMA	TC > 1%	Worse prognosis
Humphries et al. [17]	≥ 109, various types	SP142	TMA	> 1%, epithelial and lymphoid cells	Better prognosis
Karnik et al. [18]	136, ductal (primary and metastasis)	22C3, SP263	WSS (biopsies and resections)	TC ≥ 1%	Not performed
Li et al. [19]	191, HER2 positive, no NACT	22C3, 28–8	TMA	TC ≥ 1%; IC, cutoff not defined	Better prognosis
Pelekanou et al. [20]	163, HER2 negative, locally advanced, or IBC (120, pretreatment; 43, post NACT)	22C3	WSS	Either tumor or stromal cells ≥ 1%	Not associated (but better pCR)
Downes et al. [21]	30, not specified	22C3, SP142, SP263	TMA	22C3: CPS ≥ 1; SP142: IC ≥ 1%; SP263: cutoff not defined	Not performed
Noske et al. [22]	1318, various types, all node-positive	SP263	TMA	TC ≥ 1%; IC ≥ 1%	Not associated
Van Berckelaer et al. [23]	349 (207, pretreatment IBC; 142, non-IBC)	SP142	WSS (biopsies)	TC, IC (categorized based on %)	Not associated (but better pCR)

*IBC* inflammatory carcinoma, *NACT* neoadjuvant chemotherapy, *TC* tumor cell, *IC* immune cell, *CPS* combined positive score, *TMA* tissue microarray, *WSS* whole slide section, *pCR* pathologic complete response

In the assessment of PD-L1 expression in solid tumors, like IHC antibody and scoring method, cutoff value is another variable that may lead to divergent results. There were several reasons for selecting 1% as the cutoff point in our study in order to compare the three scoring methods. A meta-analysis of 20 clinical trials of anti-PD-1/PD-L1 therapy in melanoma, non-small cell lung cancer, and renal cell carcinoma patients noted that 1% was among the most frequently used cutoff values for PD-L1 [35]. One percent was also used as the cutoff in the majority of recent studies of PD-L1 expression in breast cancer using FDA-approved antibodies [16–23] (Table 9). In recent published TNBC clinical trials with pembrolizumab and atezolizumab, a Combined Positive Score of 1 (the equivalent of 1% TCIC in our study) and a 1% IC score, respectively, were the cutoffs used to evaluate PD-L1 expression, and the latter was included as the cutoff in selecting patients in the FDA approval of the drug [13, 14]. With ≥ 1% considered positive in our study, in the TNBC group, the positive rate for PD-L1 expression was 31% by IC, somewhat lower than the 41% rate of positivity reported in the IMpassion 130 trial [14]. Pre-selection of the cohorts may have played a role to lead to this difference. In our cohort, all patients had surgical resection of the primary tumor, and most did not have metastasis, whereas the IMpassion 130 trial focused on metastatic or unresectable locally advanced TNBC. Based on our finding of PD-L1 being an indicator for worse prognosis, it is reasonable to postulate that the IMpassion 130 trial may have selected patients who were more likely to have PD-L1 expression. Also, it has been shown that PD-L1 expression in breast cancer is focal or patchy [36]; therefore, the use of TMA in our study may partially explain the lower positivity than that reported in the IMpassion 130 trial. In addition, the difference may be due to our small sample size, interobserver variation, or differences in the antibodies.

Our results on sTILs showed a trend for better recurrence free-survival with higher sTIL level (≥ 10%) in the TNBC group without NACT. This result is consistent with findings by others showing that higher TIL is a good prognostic indicator [37, 38]. Because PD-L1 was associated with sTILs in many subgroups in our study, and because PD-L1 and sTILs had significant associations or trends with survival, the prognostic role of PD-L1 in subgroups according to sTIL level was further examined. In the subgroups with high sTIL levels, positive PD-L1 by TC showed a trend for worse distant metastasis-free survival in the TNBC group without NACT (Fig. 4c), and positive PD-L1 by IC showed a trend for worse distant metastasis-free survival in the TNBC group with NACT (Fig. 4d). There was no association or trend found in the subgroups with low sTIL levels. The trends in the post-NACT TNBC subgroup with or without further grouping by sTIL level (Figs. 3 and 4) are particularly interesting. It is well known that among TNBC patients who do not experience pathologic complete response, not all patients have relapse. It has also been shown that in TNBC patients, the presence of high TIL levels in residual disease after NACT is associated with better prognosis [39, 40]. Although our sample size was small and further investigation is necessary to confirm our findings, our results suggest that PD-L1 could serve as a marker to select TNBC patients for further treatment after NACT, and it may further stratify those with high sTILs in residual tumors in terms of prognosis.

The urgent clinical need for effective immunotherapy in treating breast cancer, especially TNBC, is mirrored by rapid new advances in this area. Studies such as that presented by Rugo et al. at the 2019 European Society for Medical Oncology (ESMO) Congress have compared PD-L1 expression in TNBC between different PD-L1 assays using different scoring methods. In that study (abstract LBA20), using the SP142 assay with an IC 1% cutoff and the 22C3 assay with a Combined Positive Score 1 cutoff, 45% of TNBC patients had positive PD-L1 expression with both assays, 36% patients had positive expression with 22C3 and negative expression with SP142, and 18% had negative expression with both assays. In the current study, a high concordance (96%) between IC and TCIC was reached using the 1% cutoff with the 22C3 assay, suggesting that the difference observed in the study by Rugo et al. may be due mainly to the differences between the assays. The recent press release on KEYNOTE-355 indicated the importance of cutoff values; in that study, a Combined Positive Score of ≥ 10 identified metastatic TNBC patients who had significantly improved progression-free survival on pembrolizumab plus chemotherapy compared to chemotherapy alone (https://bit.ly/2HtT4rj; unpublished data). It is not surprising that when the cutoff value was raised to TCIC 10% in our cohort, the positive rate was much lower (10% in the entire cohort and 19% in the TNBC group) compared with the positive rate when TCIC 1% was used (20% in the entire cohort and 35% in the TNBC group). Although the goal of this study was to demonstrate how each of the three scoring methods impacts PD-L1 evaluation using one assay, our other ongoing studies aim to investigate how different assays detect PD-L1 expression. It would be interesting to compare PD-L1 expression using a TCIC 10% cutoff with 22C3 and using an IC 1% cutoff with SP142.

Our study had the limitations of a retrospective study. Because many HER2-positive and TNBC patients receive neoadjuvant therapy and show pathologic complete response, selection bias inevitably influenced the makeup of the subgroups with and without NACT in our study. Also, although the study design included two different areas of the tumor to make duplicate TMA punches, the validity of using TMA to capture PD-L1 expression in breast cancer needs to be further verified. In addition, the predictive value of PD-L1 in anti-PD1/PD-L1 therapy could not be addressed in our study since none of the patients was treated with anti-PD1/PD-L1. Furthermore, although our entire cohort was relatively large, some of the subgroups had small numbers of patients, hindering meaningful statistical analysis. Additional studies with cohorts enriched for rarer subtypes and in patient populations given anti-PD1/PD-L1 therapy may provide useful information in these regards.

The identification of the role of PD-1/PD-L1 in tumor immune escape more than a decade ago has revolutionized immunotherapy in human tumors [41, 42]. With accumulating experience, it has become conceivable that key players such as TILs, PD-1, and PD-L1 act dynamically in the process of tumor initiation and progression. Therefore, detection of PD-L1 expression by IHC in a tissue sample may provide only a glimpse of the tumor in its interaction with the immune response. Noninvasive approaches that can reflect dynamic changes of spatial and temporal PD-L1 expression in the tumor would no doubt guide more efficient treatment. To this end, positron emission tomography (PET) imaging studies to detect PD-L1 expression in vivo have shown promising advances [43, 44]. However, before such techniques are available for clinical practice, IHC remains the best assay to evaluate PD-L1 expression in human tumors. Although most patients receiving anti-PD-1/PD-L1 therapy react with only mild toxicity profiles, including skin rash, dysthyroidism, and gastrointestinal events, more severe immune-mediated side effects occur in some patients, and treatment-related deaths have been reported [1, 2, 5]. Therefore, to select patients for this treatment and avoid unnecessary adverse effects, technical issues related to PD-L1 IHC such as the one addressed in this study are of clinical importance.

## Conclusions

Our findings indicate that the three scoring methods, with a 1% cutoff, are different in their sensitivity for detecting PD-L1 and their associations with clinicopathologic factors and clinical outcomes. We have shown that scoring by TCIC is the most sensitive way to identify PD-L1-positive breast cancer. In a setting where the desire is to include as many patients as possible for a clinical trial, this score may be the most useful. Alternatively, one can use the combination of the TC score and IC score to reach almost the same sensitivity, although this comparability may be dependent on the selected cutoff values of the individual scores. On the other hand, when PD-L1 expression is assessed as a prognostic marker, our study suggests that PD-L1 is associated with worse clinical outcome, most often shown by the IC score; however, the other scores may also have clinical implications in some subgroups. Beyond the population untreated with immune checkpoint inhibitors studied here, the predictive values of these scoring methods for anti-PD-1/PD-L1 therapy are deferred to large clinical trials.

## Supplementary information

**Additional file 1: Table S1.** Association of PD-L1 staining with clinicopathologic factors in estrogen receptor/progesterone receptor positive patients without neoadjuvant chemotherapy. **Table S2.** Association of PD-L1 staining with clinicopathologic factors in estrogen receptor/progesterone receptor positive patients with neoadjuvant chemotherapy. **Table S3.** Association of PD-L1 staining with clinicopathologic factors in HER2 positive patients without neoadjuvant chemotherapy. **Table S4.** Association of PD-L1 staining with clinicopathologic factors in HER2 positive patients with neoadjuvant chemotherapy. **Table S5.** Summary of multivariate analysis in estrogen receptor/progesterone receptor positive patients without neoadjuvant chemotherapy showing the odds ratio (95% confidence interval) of variables significantly associated with PD-L1 scoring methods and sTIL level. **Figure S1.** Kaplan-Meier plots of recurrence-free survival between tumors with higher stromal tumor-infiltrating lymphocyte level (≥10%) and lower stromal tumor-infiltrating lymphocyte in the triple negative group without neoadjuvant chemotherapy.

## Data Availability

All data generated or analyzed during this study are included in this published article and its supplementary information files.

## Abbreviations

ER: Estrogen receptor
FDA: Food and Drug Administration
IDC: Invasive ductal carcinoma
ILC: Invasive lobular carcinoma
NACT: Neoadjuvant chemotherapy
PD-1: Programmed death-1
PD-L1: Programmed death-ligand 1
PR: Progesterone receptor
RCB: Residual cancer burden
sTIL: Stromal tumor infiltrating lymphocytes
TMA: Tissue microarrays
TNBC: Triple-negative breast cancer
