# Supplementary material for: Comparison of three scoring methods using the FDA-approved 22C3 immunohistochemistry assay to evaluate PD-L1 expression in breast cancer and their association with clinicopathologic factors
Source: Breast Cancer Res. 2020 Jun 23;22:69. doi: 10.1186/s13058-020-01303-9 (PMC7310491; doi:10.1186/s13058-020-01303-9)
Supplement: Supplementary file 1 — Additional file 1: Table S1. Association of PD-L1 staining with clinicopathologic factors in estrogen receptor/progesterone receptor positive patients without neoadjuvant chemotherapy. Table S2. Association of PD-L1 staining with clinicopathologic factors in estrogen receptor/progesterone receptor positive patients with neoadjuvant chemotherapy. Table S3. Association of PD-L1 staining with clinicopathologic factors in HER2 positive patients without neoadjuvant chemotherapy. Table S4. Association of PD-L1 staining with clinicopathologic factors in HER2 positive patients with neoadjuvant chemotherapy. Table S5. Summary of multivariate analysis in estrogen receptor/progesterone receptor positive patients without neoadjuvant chemotherapy showing the odds ratio (95% confidence interval) of variables significantly associated with PD-L1 scoring methods and sTIL level. Figure S1. Kaplan-Meier plots of recurrence-free survival between tumors with higher stromal tumor-infiltrating lymphocyte level (≥10%) and lower stromal tumor-infiltrating lymphocyte in the triple negative group without neoadjuvant chemotherapy. [file 13058_2020_1303_MOESM1_ESM.docx]

Supplementary Table S1 Association of PD-L1 staining with clinicopathologic factors in estrogen receptor/progesterone receptor positive patients without neoadjuvant chemotherapy

|  | **TC** | | | **TCIC** | | | **IC** | | | **sTIL level** | | | |
| --- | --- | --- | --- | --- | --- | --- | --- | --- | --- | --- | --- | --- | --- |
| **Factor** | **Negative  N(%)** | **Positive  N(%)** | **Fisher**  **P value** | **Negative  N(%)** | **Positive  N(%)** | **Fisher**  **P value** | **Negative  N(%)** | **Positive  N(%)** | **Fisher**  **P value** | **Low  N(%)** | **High  N(%)** | **Fisher**  **P value** |  |
| Age | [N=247] | |  | [N=247] | |  | [N=247] | |  | [N=250] | |  |  |
| <50 years | 57(90) | 6(10) | 1 | 50(79) | 13(21) | 0.3214 | 52(83) | 11(17) | 0.1888 | 49(74) | 17(26) | **0.0179** |  |
| ≥50 years | 167(91) | 17(9) |  | 157(85) | 27(15) |  | 164(89) | 20(11) |  | 161(88) | 23(13) |  |  |
| Race/ethnicity | [N=247] | |  | [N=247] | |  | [N=247] | |  | [N=250] | |  |  |
| Black | 16(80) | 4(20) | 0.0658 | 11(55) | 9(45) | **0.0013** | 12(60) | 8(40) | **0.0004** | 12(63) | 7(37) | **0.0372** |  |
| White+Latino | 194(92) | 16(8) |  | 183(87) | 27(13) |  | 191(91) | 19(9) |  | 184(86) | 30(14) |  |  |
| Others | 14(82) | 3(18) |  | 13(76) | 4(24) |  | 13(76) | 4(24) |  | 14(82) | 3(18) |  |  |
| Histologic subtype | [N=247] | |  | [N=247] | |  | [N=247] | |  | [N=250] | |  |  |
| IDC | 180(91) | 18(9) | 0.906 | 164(83) | 34(17) | 0.6903 | 169(85) | 29(15) | 0.1083 | 161(81) | 38(19) | **0.0237** |  |
| ILC | 33(89) | 4(11) |  | 33(89) | 4(11) |  | 36(97) | 1(3) |  | 35(95) | 2(5) |  |  |
| Metaplastic | 0(0) | 0(0) |  | 0(0) | 0(0) |  | 0(0) | 0(0) |  | 0(0) | 0(0) |  |  |
| Mixed IDC/ILC | 11(92) | 1(8) |  | 10(83) | 2(17) |  | 11(92) | 1(8) |  | 14(100) | 0(0) |  |  |
| Histologic grade | [N=247] | |  | [N=247] | |  | [N=247] | |  | [N=250] | |  |  |
| 1+2 | 189(95) | 9(5) | **<0.0001** | 178(90) | 20(10) | **<0.0001** | 184(93) | 14(7) | **<0.0001** | 184(91) | 18(9) | **<0.0001** |  |
| 3 | 35(71) | 14(29) |  | 29(59) | 20(41) |  | 32(65) | 17(35) |  | 26(54) | 22(46) |  |  |
| sTIL level | [N=244] | |  | [N=244] | |  | [N=244] | |  | NA | |  |  |
| <10% | 195(95) | 10(5) | **<0.0001** | 188(92) | 17(8) | **<0.0001** | 195(95) | 10(5) | **<0.0001** |  |  |  |  |
| ≥10% | 26(67) | 13(33) |  | 17(44) | 22(56) |  | 18(46) | 21(54) |  |  |  |  |  |
| Tumor size | [N=247] | |  | [N=247] | |  | [N=247] | |  | [N=250] | |  |  |
| ≤2cm | 128(91) | 13(9) | 0.798 | 117(83) | 24(17) | 0.3084 | 121(86) | 20(14) | 0.4964 | 125(86) | 20(14) | 0.0912 |  |
| >2 to 5cm | 75(89) | 9(11) |  | 69(82) | 15(18) |  | 74(88) | 10(12) |  | 65(77) | 19(23) |  |  |
| >5cm | 21(95) | 1(5) |  | 21(95) | 1(5) |  | 21(95) | 1(5) |  | 20(95) | 1(5) |  |  |
| pN | [N=245] | |  | [N=245] | |  | [N=245] | |  | [N=248] | |  |  |
| (y)pN0 | 148(92) | 13(8) | 0.1514 | 136(84) | 25(16) | 0.6107 | 140(87) | 21(13) | 0.3148 | 137(85) | 25(15) | 0.9537 |  |
| (y)pN1 | 65(92) | 6(8) |  | 60(85) | 11(15) |  | 63(89) | 8(11) |  | 59(82) | 13(18) |  |  |
| (y)pN2 | 6(86) | 1(14) |  | 6(86) | 1(14) |  | 7(100) | 0(0) |  | 7(88) | 1(13) |  |  |
| (y)pN3 | 4(67) | 2(33) |  | 4(67) | 2(33) |  | 4(67) | 2(33) |  | 5(83) | 1(17) |  |  |
| pM | [N=245] | |  | [N=245] | |  | [N=245] | |  | [N=248] | |  |  |
| 0 | 221(91) | 21(9) | 0.2468 | 204(84) | 38(16) | 0.407 | 211(87) | 31(13) | 1 | 205(84) | 40(16) | 1 |  |
| 1 | 2(67) | 1(33) |  | 2(67) | 1(33) |  | 3(100) | 0(0) |  | 3(100) | 0(0) |  |  |
| pStage | [N=245] | |  | [N=245] | |  | [N=245] | |  | [N=248] | |  |  |
| I | 121(92) | 10(8) | 0.134 | 113(86) | 18(14) | 0.4224 | 117(89) | 14(11) | 0.6701 | 117(87) | 17(13) | 0.2297 |  |
| II | 81(91) | 8(9) |  | 72(81) | 17(19) |  | 75(84) | 14(16) |  | 68(77) | 20(23) |  |  |
| III | 18(82) | 4(18) |  | 18(82) | 4(18) |  | 19(86) | 3(14) |  | 20(87) | 3(13) |  |  |
| IV | 2(67) | 1(33) |  | 2(67) | 1(33) |  | 3(100) | 0(0) |  | 3(100) | 0(0) |  |  |

IDC: invasive ductal carcinoma; ILC: invasive lobular carcinoma; sTIL: stromal tumor infiltrating lymphocytes.

Supplementary Table S2 Association of PD-L1 staining with clinicopathologic factors in estrogen receptor/progesterone receptor positive patients with neoadjuvant chemotherapy

|  | **TC** | | | **TCIC** | | | | **IC** | | | | **sTIL level** | | |
| --- | --- | --- | --- | --- | --- | --- | --- | --- | --- | --- | --- | --- | --- | --- |
| **Factor** | **Negative  N(%)** | **Positive  N(%)** | **Fisher**  **P value** | **Negative  N(%)** | **Positive  N(%)** | | **Fisher**  **P value** | | **Negative  N(%)** | **Positive  N(%)** | **Fisher**  **P value** | **Low  N(%)** | **High  N(%)** | **Fisher**  **P value** |
| Age | [N=93] | |  | [N=87] | |  | | [N=87] | | |  | [N=95] | |  |
| <50 years | 31(94) | 2(6) | 0.2862 | 28(90) | 3(10) | | 1 | | 29(94) | 2(6) | 1 | 26(76) | 8(24) | 0.254 |
| ≥50 years | 59(98) | 1(2) |  | 51(91) | 5(9) | |  | | 51(91) | 5(9) |  | 53(87) | 8(13) |  |
| Race/ethnicity | [N=93] | |  | [N=87] | |  | | [N=87] | | |  | [N=95] | |  |
| Black | 15(88) | 2(12) | **0.0225** | 14(82) | 3(18) | | 0.2751 | | 15(88) | 2(12) | 0.5442 | 13(76) | 4(24) | 0.6672 |
| White+Latino | 66(100) | 0(0) |  | 57(93) | 4(7) | |  | | 57(93) | 4(7) |  | 57(84) | 11(16) |  |
| Others | 9(90) | 1(10) |  | 8(89) | 1(11) | |  | | 8(89) | 1(11) |  | 9(90) | 1(10) |  |
| Histologic subtype | [N=93] | |  | [N=87] | |  | | [N=87] | | |  | [N=95] | |  |
| IDC | 70(96) | 3(4) | 1 | 60(88) | 8(12) | | 0.5545 | | 61(90) | 7(10) | 0.5317 | 60(80) | 15(20) | 0.5014 |
| ILC | 16(100) | 0(0) |  | 15(100) | 0(0) | |  | | 15(100) | 0(0) |  | 15(94) | 1(6) |  |
| Metaplastic | 1(100) | 0(0) |  | 1(100) | 0(0) | |  | | 1(100) | 0(0) |  | 1(100) | 0(0) |  |
| Mixed IDC/ILC | 3(100) | 0(0) |  | 3(100) | 0(0) | |  | | 3(100) | 0(0) |  | 3(100) | 0(0) |  |
| Histologic grade^a^ | [N=93] | |  | [N=87] | |  | | [N=87] | | |  | [N=95] | |  |
| 1+2 | 63(100) | 0(0) | **0.0313** | 57(95) | 3(5) | | 0.101 | | 57(95) | 3(5) | 0.1965 | 57(88) | 8(12) | 0.1375 |
| 3 | 27(90) | 3(10) |  | 22(81) | 5(19) | |  | | 23(85) | 4(15) |  | 22(73) | 8(27) |  |
| sTIL level | [N=93] | |  | [N=87] | |  | | [N=87] | | |  | NA | |  |
| <10% | 77(100) | 0(0) | **0.0043** | 70(97) | 2(3) | | **0.0002** | | 70(97) | 2(3) | **0.0014** |  |  |  |
| ≥10% | 13(81) | 3(19) |  | 9(60) | 6(40) | |  | | 10(67) | 5(33) |  |  |  |  |
| Tumor size | [N=93] | |  | [N=87] | |  | | [N=87] | | |  | [N=95] | |  |
| ≤2cm | 14(93) | 1(7) | 0.3825 | 11(79) | 3(21) | | 0.1213 | | 11(79) | 3(21) | 0.1327 | 11(69) | 5(31) | 0.2305 |
| >2 to 5cm | 40(95) | 2(5) |  | 38(90) | 4(10) | |  | | 39(93) | 3(7) |  | 36(84) | 7(16) |  |
| >5cm | 36(100) | 0(0) |  | 30(97) | 1(3) | |  | | 30(97) | 1(3) |  | 32(89) | 4(11) |  |
| ypN | [N=93] | |  | [N=87] | |  | | [N=87] | | |  | [N=95] | |  |
| (y)pN0 | 20(91) | 2(9) | 0.4098 | 18(82) | 4(18) | | 0.1694 | | 19(86) | 3(14) | 0.3442 | 17(77) | 5(23) | 0.8713 |
| (y)pN1 | 37(97) | 1(3) |  | 31(89) | 4(11) | |  | | 31(89) | 4(11) |  | 34(85) | 6(15) |  |
| (y)pN2 | 17(100) | 0(0) |  | 16(100) | 0(0) | |  | | 16(100) | 0(0) |  | 14(82) | 3(18) |  |
| (y)pN3 | 16(100) | 0(0) |  | 14(100) | 0(0) | |  | | 14(100) | 0(0) |  | 14(88) | 2(13) |  |
| pM | [N=93] | |  | [N=87] | |  | | [N=87] | | |  | [N=95] | |  |
| 0 | 84(97) | 3(3) | 1 | 75(90) | 8(10) | | 1 | | 76(92) | 7(8) | 1 | 73(82) | 16(18) | 0.5851 |
| 1 | 6(100) | 0(0) |  | 4(100) | 0(0) | |  | | 4(100) | 0(0) |  | 6(100) | 0(0) |  |
| RCB category | [N=92] | |  | [N=86] | |  | | [N=86] | | |  | [N=94] | |  |
| I | 2(100) | 0(0) | 0.6374 | 2(100) | 0(0) | | 1 | | 2(100) | 0(0) | 1 | 2(100) | 0(0) | 1 |
| II | 42(95) | 2(5) |  | 37(90) | 4(10) | |  | | 38(93) | 3(7) |  | 38(83) | 8(17) |  |
| III | 45(98) | 1(2) |  | 39(91) | 4(9) | |  | | 39(91) | 4(9) |  | 38(83) | 8(17) |  |

^a^Histologic grade for post-treatment tumors was based on pre-treatment grade.

IDC: invasive ductal carcinoma; ILC: invasive lobular carcinoma; sTIL: stromal tumor infiltrating lymphocytes; RCB: residual cancer burden.

Supplementary Table S3 Association of PD-L1 staining with clinicopathologic factors in HER2 positive patients without neoadjuvant chemotherapy

|  | **TC [N=31]** | | | **TCIC [N=31]** | | | **IC [N=31]** | | | **sTIL level [N=33]** | | |
| --- | --- | --- | --- | --- | --- | --- | --- | --- | --- | --- | --- | --- |
| **Factor** | **Negative N(%)** | **Positive N(%)** | **Fisher P value** | **Negative N(%)** | **Positive N(%)** | **Fisher P value** | **Negative N(%)** | **Positive N(%)** | **Fisher P value** | **Low N(%)** | **High N(%)** | **Fisher P value** |
|  |  |  |  |  |  |  |  |  |  |  |  |  |
| Age |  | |  |  | |  |  | |  |  | |  |
| <50 years | 10(77) | 3(23) | 0.6254 | 6(46) | 7(54) | 0.2623 | 7(54) | 6(46) | 0.1143 | 4(29) | 10(71) | **0.0366** |
| ≥50 years | 16(89) | 2(11) |  | 13(72) | 5(28) |  | 15(83) | 3(17) |  | 13(68) | 6(32) |  |
| Race/ethnicity |  | |  |  | |  |  | |  |  | |  |
| Black | 4(100) | 0(0) | 1 | 2(50) | 2(50) | 1 | 2(50) | 2(50) | 0.69 | 1(20) | 4(80) | 0.1003 |
| White+Latino | 21(81) | 5(19) |  | 16(62) | 10(38) |  | 19(73) | 7(27) |  | 16(59) | 11(41) |  |
| Others | 1(100) | 0(0) |  | 1(100) | 0(0) |  | 1(100) | 0(0) |  | 0(0) | 1(100) |  |
| Histologic type |  | |  |  | |  |  | |  |  | |  |
| IDC | 25(83) | 5(17) | 1 | 18(60) | 12(40) | 1 | 21(70) | 9(30) | 1 | 16(50) | 16(50) | 1 |
| ILC | 1(100) | 0(0) |  | 1(100) | 0(0) |  | 1(100) | 0(0) |  | 1(100) | 0(0) |  |
| Histologic grade |  | |  |  | |  |  | |  |  | |  |
| 1+2 | 13(93) | 1(7) | 0.3445 | 11(79) | 3(21) | 0.1378 | 13(93) | 1(7) | **0.0207** | 9(60) | 6(40) | 0.4905 |
| 3 | 13(76) | 4(24) |  | 8(47) | 9(53) |  | 9(53) | 8(47) |  | 8(44) | 10(56) |  |
| sTIL level |  | |  |  | |  |  | |  | NA | |  |
| <10% | 14(88) | 2(13) | 0.6539 | 14(88) | 2(13) | **0.0032** | 16(100) | 0(0) | **0.0002** |  |  |  |
| ≥10% | 12(80) | 3(20) |  | 5(33) | 10(67) |  | 6(40) | 9(60) |  |  |  |  |
| ER |  | |  |  | |  |  | |  | [N=33] | |  |
| Negative | 4(50) | 4(50) | **0.0098** | 4(44) | 5(56) | 0.2534 | 6(67) | 3(33) | 1 | 5(56) | 4(44) | 1 |
| Positive | 22(96) | 1(4) |  | 15(68) | 7(32) |  | 16(73) | 6(27) |  | 12(50) | 12(50) |  |
| PR |  | |  |  | |  |  | |  | [N=33] | |  |
| Negative | 8(62) | 5(38) | **0.0076** | 4(29) | 10(71) | **0.0011** | 7(50) | 7(50) | **0.0439** | 6(40) | 9(60) | 0.3028 |
| Positive | 18(100) | 0(0) |  | 15(88) | 2(12) |  | 15(88) | 2(12) |  | 11(61) | 7(39) |  |
| Tumor size |  | |  |  | |  |  | |  |  | |  |
| ≤2cm | 15(88) | 2(12) | 0.6878 | 13(72) | 5(28) | 0.1865 | 14(78) | 4(22) | 0.587 | 10(56) | 8(44) | 0.857 |
| >2 to 5cm | 10(77) | 3(23) |  | 6(50) | 6(50) |  | 7(58) | 5(42) |  | 7(50) | 7(50) |  |
| >5cm | 1(100) | 0(0) |  | 0(0) | 1(100) |  | 1(100) | 0(0) |  | 0(0) | 1(100) |  |
| pN |  | |  |  | |  |  | |  |  | |  |
| (y)pN0 | 19(79) | 5(21) | 0.6873 | 16(64) | 9(36) | 0.3757 | 18(72) | 7(28) | 0.541 | 14(56) | 11(44) | 0.7326 |
| (y)pN1 | 5(100) | 0(0) |  | 3(75) | 1(25) |  | 3(75) | 1(25) |  | 3(50) | 3(50) |  |
| (y)pN2 | 1(100) | 0(0) |  | 0(0) | 1(100) |  | 0(0) | 1(100) |  | 0(0) | 1(100) |  |
| (y)pN3 | 1(100) | 0(0) |  | 0(0) | 1(100) |  | 1(100) | 0(0) |  | 0(0) | 1(100) |  |
| pM |  | |  |  | |  |  | |  |  | |  |
| 0 | 26(84) | 5(14) | NA | 19(61) | 12(39) | NA | 22(71) | 9(29) | NA | 17(52) | 16(48) | NA |
| 1 | 0(0) | 0(0) |  | 0(0) | 0(0) |  | 0(0) | 0(0) |  | 0(0) | 0(0) |  |
| pStage |  | |  |  | |  |  | |  |  | |  |
| I | 14(82) | 3(18) | 1 | 13(72) | 5(28) | 0.1285 | 15(83) | 3(17) | 0.2091 | 11(61) | 7(39) | 0.2594 |
| II | 10(83) | 2(17) |  | 6(55) | 5(45) |  | 6(55) | 5(45) |  | 6(46) | 7(54) |  |
| III | 2(100) | 0(0) |  | 0(0) | 2(100) |  | 1(50) | 1(50) |  | 0(0) | 2(100) |  |
| IV | 0(0) | 0(0) |  | 0(0) | 0(0) |  | 0(0) | 0(0) |  | 0(0) | 0(0) |  |

IDC: invasive ductal carcinoma; ILC: invasive lobular carcinoma; sTIL: stromal tumor infiltrating lymphocytes; ER: estrogen receptor; PR: progesterone receptor.

Supplementary Table S4 Association of PD-L1 staining with clinicopathologic factors in HER2 positive patients with neoadjuvant chemotherapy

|  | **TC [N=11]** | | | **TCIC [N=10]** | | | **IC [N=10]** | | | **sTIL level [N=13]** | | |
| --- | --- | --- | --- | --- | --- | --- | --- | --- | --- | --- | --- | --- |
| **Factor** | **Negative N(%)** | **Positive N(%)** | **Fisher P value** | **Negative N(%)** | **Positive N(%)** | **Fisher P value** | **Negative N(%)** | **Positive N(%)** | **Fisher P value** | **Low N(%)** | **High N(%** | **Fisher P value** |
|  |  |  |  |  |  |  |  |  |  |  |  |  |
| Age |  | |  |  | |  |  | |  |  | |  |
| <50 years | 5(100) | 0(0) | NA | 5(100) | 0(0) | 1 | 5(100) | 0(0) | 1 | 5(83) | 1(17) | 1 |
| ≥50 years | 6(100) | 0(0) |  | 4(80) | 1(20) |  | 4(80) | 1(20) |  | 6(86) | 1(14) |  |
| Race/ethnicity |  | |  |  | |  |  | |  |  | |  |
| Black | 2(100) | 0(0) | NA | 2(100) | 0(0) | 0.1 | 2(100) | 0(0) | 0.1 | 2(100) | 0(0) | 0.5385 |
| White+Latino | 8(100) | 0(0) |  | 7(100) | 0(0) |  | 7(100) | 0(0) |  | 8(89) | 1(11) |  |
| Others | 1(100) | 0(0) |  | 0(0) | 1(100) |  | 0(0) | 1(100) |  | 1(50) | 1(50) |  |
| Histologic type |  | |  |  | |  |  | |  |  | |  |
| IDC | 10(100) | 0(0) | NA | 8(89) | 1(11) | 1 | 8(89) | 1(11) | 1 | 10(83) | 2(17) | 1 |
| ILC | 1(100) | 0(0) |  | 1(100) | 0(0) |  | 1(100) | 0(0) |  | 1(100) | 0(0) |  |
| Histologic grade^a^ |  | |  |  | |  |  | |  |  | |  |
| 1+2 | 4(100) | 0(0) | NA | 4(100) | 0(0) | 1 | 4(100) | 0(0) | 1 | 6(100) | 0(0) | 0.4615 |
| 3 | 7(100) | 0(0) |  | 5(83) | 1(17) |  | 5(83) | 1(17) |  | 5(71) | 2(29) |  |
| sTILlevel |  | |  |  | |  |  | |  | NA | |  |
| <10% | 9(100) | 0(0) | NA | 8(100) | 0(0) | 0.2 | 8(100) | 0(0) | 0.2 |  |  |  |
| ≥10% | 2(100) | 0(0) |  | 1(50) | 1(50) |  | 1(50) | 1(50) |  |  |  |  |
| ER |  | |  |  | |  |  | |  |  | |  |
| Negative | 2(100) | 0(0) | NA | 1(100) | 0(0) | 1 | 1(100) | 0(0) | 1 | 2(100) | 0(0) | 1 |
| Positive | 9(100) | 0(0) |  | 8(89) | 1(11) |  | 8(89) | 1(11) |  | 9(82) | 2(18) |  |
| PR |  | |  |  | |  |  | |  |  | |  |
| Negative | 4(100) | 0(0) | NA | 3(100) | 0(0) | 1 | 3(100) | 0(0) | 1 | 4(100) | 0(0) | 1 |
| Positive | 7(100) | 0(0) |  | 6(86) | 1(14) |  | 6(86) | 1(14) |  | 7(78) | 2(22) |  |
| Tumor size |  | |  |  | |  |  | |  |  | |  |
| ≤2cm | 3(100) | 0(0) | NA | 2(100) | 0(0) | 1 | 2(100) | 0(0) | 1 | 4(100) | 0(0) | 0.4615 |
| >2 to 5cm | 6(100) | 0(0) |  | 5(83) | 1(17) |  | 5(83) | 1(17) |  | 4(67) | 2(33) |  |
| >5cm | 2(100) | 0(0) |  | 2(100) | 0(0) |  | 2(100) | 0(0) |  | 3(100) | 0(0) |  |
| ypN |  | |  |  | |  |  | |  |  | |  |
| (y)pN0 | 5(100) | 0(0) | NA | 4(100) | 0(0) | 0.3 | 4(100) | 0(0) | 0.3 | 7(100) | 0(0) | **0.0385** |
| (y)pN1 | 2(100) | 0(0) |  | 1(50) | 1(50) |  | 1(50) | 1(50) |  | 1(50) | 1(50) |  |
| (y)pN2 | 1(100) | 0(0) |  | 1(100) | 0(0) |  | 1(100) | 0(0) |  | 0(0) | 1(100) |  |
| (y)pN3 | 3(100) | 0(0) |  | 3(100) | 0(0) |  | 3(100) | 0(0) |  | 3(100) | 0(0) |  |
| pM |  | |  |  | |  |  | |  |  | |  |
| 0 | 10(100) | 0(0) | NA | 8(89) | 1(11) | 1 | 8(89) | 1(11) | 1 | 10(83) | 2(17) | 1 |
| 1 | 1(100) | 0(0) |  | 1(100) | 0(0) |  | 1(100) | 0(0) |  | 1(100) | 0(0) |  |
| RCB category |  | |  |  | |  |  | |  |  | |  |
| I | 1(100) | 0(0) | NA | 1(100) | 0(0) | 1 | 1(100) | 0(0) | 1 | 2(100) | 0(0) | 0.2692 |
| II | 5(100) | 0(0) |  | 4(100) | 0(0) |  | 4(100) | 0(0) |  | 6(100) | 0(0) |  |
| III | 5(100) | 0(0) |  | 4(80) | 1(20) |  | 4(80) | 1(20) |  | 3(60) | 2(40) |  |

^a^Histologic grade for post-treatment tumors was based on pre-treatment grade.

IDC: invasive ductal carcinoma; ILC: invasive lobular carcinoma; sTIL: stromal tumor infiltrating lymphocytes; ER: estrogen receptor; PR: progesterone receptor; RCB: residual cancer burden.

Supplementary Table S5 Summary of multivariate analysis in estrogen receptor/progesterone receptor positive patients without neoadjuvant chemotherapy showing the odds ratio (95% confidence interval) of variables significantly associated with PD-L1 scoring methods and sTIL level

| **Factor** | | **TC** | **TCIC** | **IC** | **sTIL level** |
| --- | --- | --- | --- | --- | --- |
| Age | |  |  |  |  |
| ≥50 vs. <50 years | |  |  |  | 0.40 (0.18, 0.87)^a^ |
| Race/ethnicity | |  |  |  |  |
|  | White+Latino vs. Black |  |  | 0.16 (0.04, 0.60)^b^ |  |
|  | Others vs. Black |  |  | NS |  |
| Histologic grade | |  |  |  |  |
| 3 vs. 1+2 | | 4.60 (1.66,12.73)^b^ | 2.631 (1.10, 6.31)^a^ |  | 8.760 (4.09, 18.78)^c^ |
| sTIL level | |  |  |  |  |
| ≥10% vs. <10% | | 5.25 (1.89, 14.58)^b^ | 9.977 (4.21, 23.65)^c^ | 22.63 (8.76, 58.45)^c^ |  |

^a^ P value <0.05-0.01.

^b^ P value <0.01-0.001.

^c^ P value <0.001.

NS: Not significant.


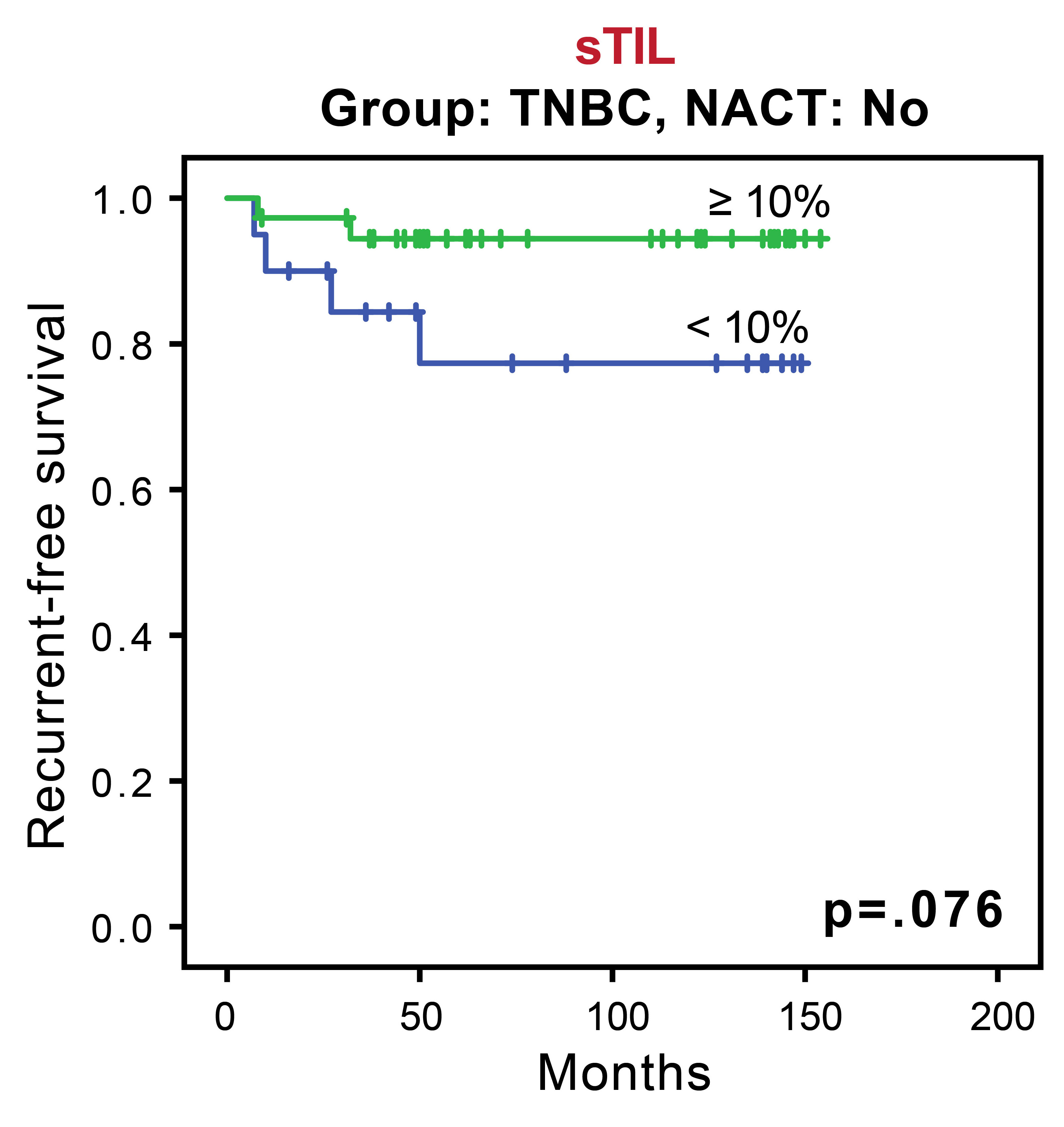


Supplementary Figure S1. Kaplan-Meier plots of recurrence-free survival between tumors with higher stromal tumor-infiltrating lymphocyte level (≥10%) and lower stromal tumor-infiltrating lymphocyte in the triple negative group without neoadjuvant chemotherapy.
